# An optimization framework for hierarchical clustering

**DOI:** 10.1093/bioadv/vbag107

**Published:** 2026-04-13

**Authors:** Gal Gilad, Roded Sharan

**Affiliations:** School of Computer Science and AI, Tel Aviv University, Tel Aviv 69978, Israel; School of Computer Science and AI, Tel Aviv University, Tel Aviv 69978, Israel

## Abstract

**Motivation:**

Hierarchical clustering is a fundamental problem in computational biology, with popular greedy heuristics such as average linkage dating back to the 1950s but no well-defined objective. Recently, a combinatorial optimization criterion for the problem was suggested by Dasgupta. While minimizing this criterion is NP-hard, the popular average linkage method serves as a strong baseline. Nevertheless, its myopic, greedy nature frequently leads to structurally suboptimal hierarchies.

**Results:**

To remedy this, we introduce a novel average-linkage-based clustering approach that combines local and global considerations by generating multiple views of the input data and learning how to blend them into an integrated similarity measure. We demonstrate that our method, DOMUS, consistently outperforms strong baselines, including a beam search heuristic, on a wide range of synthetic and classic benchmark datasets. Furthermore, we validate its real-world applicability through a rigorous benchmark on single-cell RNA sequencing data, where it compares favorably with the state-of-the-art HiDeF algorithm.

**Availability and implementation:**

The DOMUS framework is implemented in Python and freely available at https://github.com/GalGilad/DOMUS.

## 1 Introduction

Hierarchical clustering is a fundamental unsupervised learning problem that calls for grouping similar items into a nested hierarchy of clusters (Jain *et al.* 1999, Murtagh and Contreras 2012). Unlike flat partitioning methods like *K*-means, it does not require the number of clusters to be specified in advance. Instead, it produces a tree-based representation of the data, called a **dendrogram**, which specifies the relations among the clusters. This hierarchy can be generated via two main approaches: agglomerative (bottom-up), where each data point starts in its own cluster and pairs of clusters are merged as one moves up the hierarchy, or divisive (top-down), where all data points start in one cluster that is recursively split.

A key challenge in hierarchical clustering is evaluating the quality of a resulting dendrogram. Dasgupta (2016) proposed a cost function that provides a formal measure of a dendrogram’s quality based on a given similarity matrix, *W*. The intuition is that pairs of items with high similarity should be merged early in the hierarchy (when building it bottom-up), i.e. they should reside within a small subtree of the dendrogram. The total cost for a dendrogram *T* is the sum of costs over all pairs of items, formally defined as: $\text{Cost}(T)=\sum_{i<j}W_{ij}|T_{ij}|$, where $W_{ij}$ is the similarity between items *i* and *j*, and $\left|T_{ij}\right|$ is the number of leaf nodes or items in the subtree induced by the lowest common ancestor of *i* and *j*. Finding a dendrogram that minimizes this objective is known to be NP-hard (Dasgupta 2016), motivating the development of effective heuristics for the problem.

We introduce a novel framework for hierarchical clustering, which we call **DOMUS** (**D**endrogram **O**ptimization via **MU**lti-view **S**imilarity blending). Instead of directly searching the space of possible dendrograms, DOMUS approaches the problem by first constructing an optimal blended similarity matrix. The main idea is to blend diverse perspectives on the data to be clustered in order to guide the clustering algorithm toward a high-quality dendrogram. The core problem then becomes one of optimizing the parameters of this blending process. To this end, we employ a surrogate-assisted optimization algorithm.

We extensively benchmark our framework on both synthetic and real datasets. Across a diverse suite of synthetic benchmarks, DOMUS consistently finds lower cost solutions than strong baselines. This superior performance extends to classic real-world datasets, where our framework uncovers structurally distinct and more meaningful hierarchies. Finally, we validate its practical utility on a complex single-cell RNA sequencing task, showing that our general-purpose approach is competitive with state-of-the-art domain-specific network algorithms. Crucially, DOMUS produces a complete binary dendrogram, providing a comprehensive map of relationships valuable for global, exploratory data analysis.

## 1.1 Related work

Hierarchical clustering has a long history in unsupervised learning, with classical agglomerative methods such as single, complete, and average linkage remaining widely used (Sneath *et al.* 1973). Although computationally efficient and conceptually simple, such methods lack a principled global objective and are overly sensitive to highly localized similarities, often ignoring the broader global context. The introduction of Dasgupta’s cost function (Dasgupta 2016) provided a rigorous optimization criterion for hierarchical clustering, triggering a line of work on approximation algorithms and structural guarantees, including the finding that average linkage achieves a 3-approximation to the complement maximization problem (Moseley and Wang 2017), a bound that was subsequently improved by alternative algorithmic approaches (Alon *et al.* 2020). While these results establish constant-factor guarantees in worst-case settings, they do not eliminate the tendency of greedy procedures to make irreversible, myopic merge decisions on practical datasets.

To address the shortcomings of a greedy approach, several works expand the search over dendrogram space. Heuristics such as beam search maintain multiple partial merge sequences in parallel (Greenberg *et al.* 2021), while other methods generate alternative hierarchies using spectral or divisive algorithms (Ng *et al.* 2001, Kaufman and Rousseeuw 2009). However, these methods typically operate directly on tree space, modifying the merge procedure without altering the underlying similarity geometry. Newer hybrid frameworks like IDEA (Ahn *et al.* 2021) share our goal of minimizing Dasgupta’s cost, attempting to bridge this gap by combining divisive graph partitioning with an ensemble tree-building process. While IDEA optimizes the tree construction procedure directly through a specific hybridization of heuristics, DOMUS takes a representation-centric perspective: it uses these strategies as auxiliary views to mathematically reshape the similarity geometry itself, ensuring the standard linkage heuristic aligns more closely with the global objective.

Recent advances have also applied deep learning to the problem. DeepECT (Mautz *et al.* 2020) proposes a deep embedded clustering tree that jointly learns feature representations via autoencoders while iteratively growing a binary hierarchy. While effective for high-dimensional feature data, DeepECT fundamentally operates in a learned embedding space and requires raw feature inputs to optimize its reconstruction loss. This limits its applicability in domains where only pairwise similarities are available, a constraint that DOMUS—which operates purely on similarity matrices—does not share.

Our approach also relates to multi-view and ensemble clustering but differs in intent and mechanism. Standard multi-view clustering integrates multiple heterogeneous feature sources or modalities (Chaudhuri *et al.* 2009), whereas DOMUS generates diverse views from a single similarity matrix. Similarly, ensemble methods like the cluster-consensus selection framework of Huang *et al.* (2023) (and classics like Strehl and Ghosh (2003)) aim to improve robustness by filtering and aggregating clustering outputs (partitions) based on cluster-level merit scores. DOMUS, by contrast, blends diverse structural inputs prior to clustering. This strategy resembles meta-learning over similarity transformations rather than consensus voting over cluster outputs.

Finally, hierarchical clustering is central in computational biology and single-cell analysis (Eisen *et al.* 1998). Recent graph-based approaches such as HiDeF (Zheng *et al.* 2021) detect persistent multiscale communities but do not produce fully resolved binary trees. By benchmarking against HiDeF, we show that optimizing similarity structure yields competitive biological resolution while producing a complete dendrogram suitable for exploratory and lineage-scale analyses.

## 2 Methods

**Figure 1:**
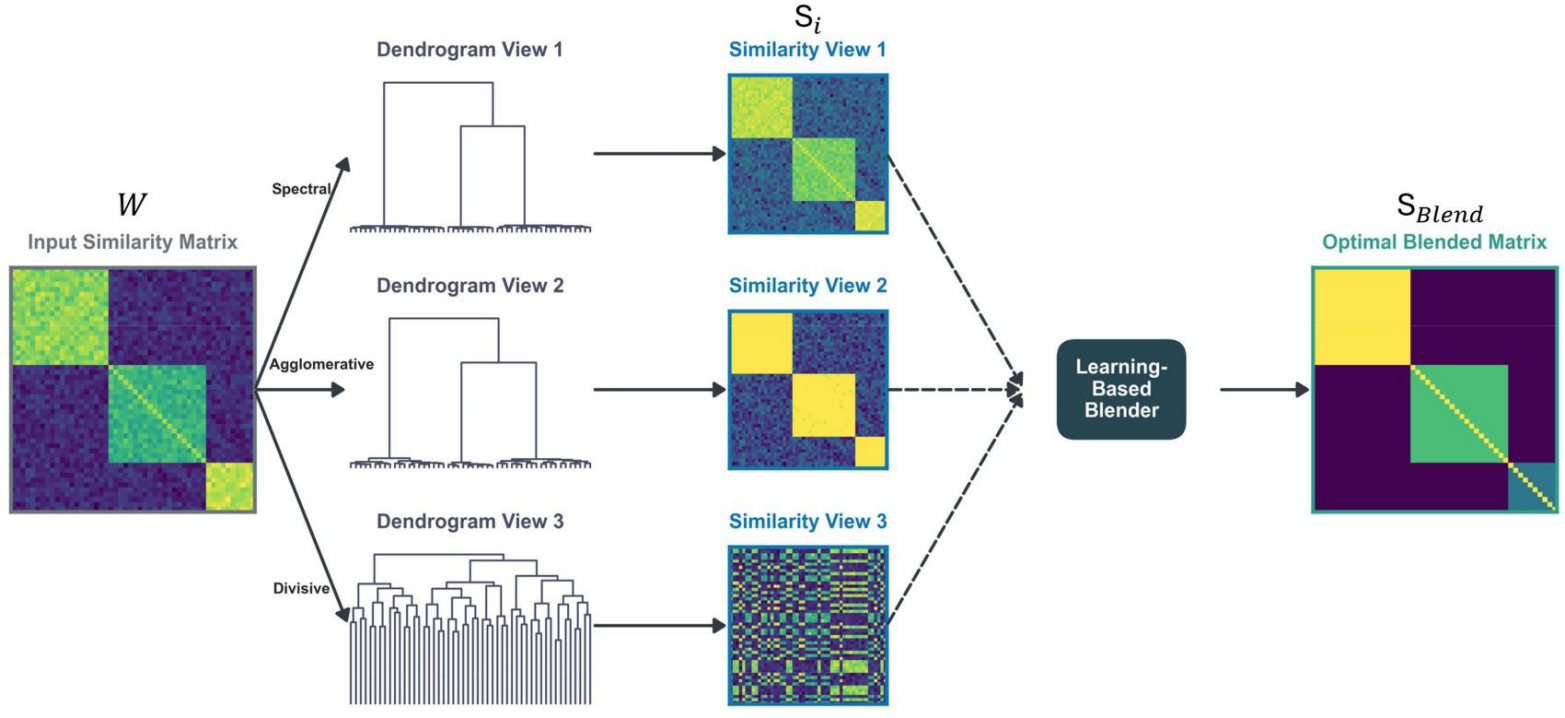
Overview of the DOMUS framework. An input similarity matrix (*W*) is used to generate multiple diverse dendrogram views (e.g. spectral, agglomerative). These are converted into new similarity matrices ($S_{i}$) which are then optimized by a learning-based blender to produce a final, optimal blended matrix ($S_{\mathrm{Blend}}$).

The DOMUS framework, depicted in Fig. 1, consists of three main stages: (i) generating multiple alternative views of the data by deriving different dendrograms from an initial similarity matrix *W*, (ii) converting each of these dendrograms into a new similarity matrix, and (iii) blending these derived matrices with the original matrix using a surrogate-assisted optimization algorithm. The final blended matrix serves as the input to a standard hierarchical clustering algorithm, with the resulting dendrogram’s quality guiding the optimization. We utilize average linkage as the final-stage algorithm in this study, as our empirical analysis identifies it as the optimal choice for minimizing the Dasgupta cost (see Section S1.1 and Fig. S1, available as supplementary data at *Bioinformatics Advances* online).

### 2.1 Derivation of alternative similarity matrices

We first generate three structurally diverse views from the initial matrix *W*: (i) Recursive Laplacian Bisection (by gap) (a global graph-based view using the Fiedler vector for iterative binary partitioning), (ii) Recursive *K*-means (a divisive view), and (iii) Centroid Linkage (an agglomerative view). This set provides a complementary portfolio of perspectives. Our full analysis for selecting this specific set and detailed descriptions of each method are available in Section S1.2 and Fig. S2, available as supplementary data at *Bioinformatics Advances* online.

Each of the resulting dendrograms is transformed into a new n×n similarity matrix *S*. The similarity $S_{ij}^{\prime }$ between two items *i* and *j* is set according to the size of the subtree they span:


(1) $$S_{ij}^{\prime }=\frac{N-|T_{ij}|}{N-2}$$

where $T_{ij}$ is the smallest subtree in the dendrogram *D* that contains both leaf nodes *i* and *j*, *N* is the total number of items, and $\left|T_{ij}\right|$ is the number of leaf nodes in that subtree. This formulation naturally assigns higher similarity to pairs of items that are grouped together in smaller subtrees, and the similarity score is normalized to the range [0,1].

### 2.2 Similarity blending and evaluation

In the final stage of the algorithm, we combine the original similarity matrix *W* and the derived similarity matrices $S_{1},S_{2},\ldots ,S_{k}$. To this end, we employ a model that allows each derived similarity matrix $S_{i}$ to have a unique power exponent $p_{si}$ while interacting with the original matrix *W*, which is shaped by a shared power $p_{w}$. The formula is:


(2) $$S_{\text{blend}}=c_{0}W+\sum_{i=1}^{k}c_{i}(W^{p_{w}}⊙S_{i}^{p_{si}})$$

where ⊙ denotes element-wise multiplication, and $c_{0},c_{1},\ldots ,c_{k}$, $p_{w}$, and $p_{s1},\ldots ,p_{sk}$ are the parameters to be optimized. The coefficients $c_{i}$ are constrained such that $c_{i}\geq 0$ and $\sum c_{i}=1$, and the power exponents are constrained to the range between 0 and 4. This range was chosen to be sufficiently broad to allow the optimizer to explore significant non-linear rescalings of the similarity space. As shown in our analysis in Section 8.2, the optimizer frequently learned exponents >2, validating the choice of a permissive upper bound.

The efficacy of the blending model is validated experimentally in Section S1.3 (Figs S3 and S4, available as supplementary data at *Bioinformatics Advances* online). Each component of this model serves a specific purpose. The **coefficients** ($c_{i}$) act as linear weights, controlling the overall influence of the original matrix versus each of the derived views. The **power exponents** ($p_{w},p_{si}$) non-linearly rescale the matrices, which can sharpen or soften the distinctions between similarity values. An exponent >1 makes high similarities more distinct, while an exponent <1 brings similarity values closer together. Finally, the **interaction term** ($W^{p_{w}}⊙S_{i}^{p_{si}}$) allows the similarity from a derived view $S_{i}$ to be contextually re-weighted by the original similarity matrix *W*. This means that if both the original data and a derived view agree that a pair of items is similar, the element-wise product amplifies this signal of agreement. Conversely, if either source considers the pair dissimilar, the resulting product is suppressed.

### 2.2.1 Surrogate-assisted optimization

Our algorithm efficiently searches the complex, likely multi-modal parameter space by using a fast surrogate model. The approach balances broad exploration with focused exploitation through three main phases (see Fig. S5 and Section S1.4, available as supplementary data at *Bioinformatics Advances* online for a full description). The process begins with an initial LHS warm-up, followed by the one-time training of a fast surrogate model. The algorithm then proceeds in iterative rounds, using the surrogate to inject promising new candidates and local hill-climbing to refine the best-known solutions. This strategy efficiently focuses the search on promising areas of the parameter space, yielding significant performance gains over a baseline (Fig. S6, available as supplementary data at *Bioinformatics Advances* online) and demonstrating consistent improvement as the iteration budget increases (Fig. S7, available as supplementary data at *Bioinformatics Advances* online).

The fitness of any given $S_{\mathrm{blend}}$ is determined by its ability to generate a high-quality hierarchical clustering. For a given $S_{\mathrm{blend}}$, we first construct a new dendrogram *T* using average linkage, a method chosen as our final-stage algorithm because, as we empirically justify in Section S1.1, available as supplementary data at *Bioinformatics Advances* online, it consistently provides the best performance for this task. The quality of this resulting dendrogram is measured by Dasgupta’s cost, which the optimization seeks to minimize. We define the “**optimal** variant” as the approach that applies this optimization procedure to minimize the Dasgupta cost directly on the original dataset. In addition, we define a “**robust** variant,” where the fitness of a candidate parameter set is determined by its average performance across *B* bootstrap samples of the data. This ensures that the optimization process rewards parameters that yield solutions resilient to data perturbations. To manage the computational load, we employ two levels of evaluation: a computationally intensive “full” evaluation using a larger number of bootstraps ($B_{\mathrm{full}}$) for assessing newly injected candidates, and a more efficient “cheap” evaluation with fewer bootstraps ($B_{\mathrm{cheap}}$) for the local hill-climbing phase. By optimizing for average performance on resampled data, this variant is explicitly guided toward solutions that are not only low-cost but also structurally stable, thereby mitigating the risk of overfitting.

### 2.2.2 Beam search heuristic

As a strong baseline, we compare DOMUS against a beam search algorithm applied to average linkage. We selected average linkage for this baseline after a comparative analysis demonstrated that it consistently produces lower cost dendrograms than beam search variants based on single, complete, or Ward linkage (see Fig. S8, available as supplementary data at *Bioinformatics Advances* online). This heuristic explores a larger portion of the search space than the standard greedy approach. At each of the n−1 merge steps, the algorithm maintains a “beam” of the *k* most promising partial clusterings. From each partial clustering in the beam, it considers the top *m* best possible next merges (based on linkage cost). All resulting new partial clusterings are generated and then pruned down to the top k to form the beam for the next step. This selection prioritizes states that have a low cumulative linkage cost and have been formed by a sequence of high-ranked merges. After all merges are complete, the final dendrogram with the lowest overall cost from the beam is selected as the solution.

Computationally, it is important to note that this heuristic is significantly more expensive than standard linkage. Unlike standard greedy linkage, which can utilize nearest-neighbor chain updates for $O(n^{2})$ efficiency, beam search maintains divergent states that preclude these local shortcuts. Consequently, its runtime complexity approaches $O(k\cdot n^{3})$ in the worst case, making it more expensive than our proposed optimization framework.

## 2.3 Data

A significant challenge in validating hierarchical clustering methods is the scarcity of real-world datasets with a gold-standard hierarchy. To address this, we designed a comprehensive three-pronged evaluation strategy. First, we use a suite of synthetic datasets to test an algorithm’s ability to recover a known ground truth. Second, we use classic flat-partitioning benchmarks to assess the pragmatic goal of grouping known classes into meaningful, low-level subtrees. Finally, we use a domain-specific single-cell RNA sequencing dataset with a known biological ontology to validate our method’s performance on a real-world hierarchical task.

## 2.3.1 Synthetic data

Our primary synthetic benchmark is a diverse set of datasets assessing performance across a broad range of data topologies. These were generated using five distinct generator types designed to test robustness to various features, including: (i) irregular Gaussian clusters, (ii) well-separated spherical clusters, (iii) non-convex shapes, (iv) nested hierarchical structures, and (v) discrete data with tied similarities. Key parameters were randomized to ensure diversity. A detailed methodology for each generator is provided in Section S2, available as supplementary data at *Bioinformatics Advances* online, and representative examples are in Fig. S9, available as supplementary data at *Bioinformatics Advances* online.

In addition, to specifically test an algorithm’s ability to navigate a deceptive cost landscape, we employ a specialized dataset known to deceive the average-linkage criterion (Charikar *et al.* 2018). This dataset is constructed to create a scenario that specifically fools the algorithm by defining two competing sets of weighted cliques. The first is a collection of small, *k*-item “decoy” cliques where internal pairwise similarities are set to a value of s+ϵ. The second is a set of larger, $k^{2}$-item “optimal” cliques where internal similarities are set to a value of *s*. Because the average similarity is maximized within the small decoy cliques, an average-linkage algorithm is predisposed to merge them, failing to identify the larger, more globally coherent optimal clusters.

## 2.3.2 Classic benchmark datasets

We use two classic datasets from the UCI Machine Learning Repository, chosen to evaluate our method’s performance on both categorical and numerical feature types. The first is the Zoo dataset, which contains 101 animals, each described by 16 categorical and numerical features. The key feature of this dataset is its well-defined ground truth, where each animal belongs to one of seven distinct classes (e.g. mammal, bird, fish). This allows for an objective evaluation of how well a clustering method can recover the known biological groupings from primarily categorical data.

The second is the Iris dataset, another standard benchmark, which contains 150 flower samples from three distinct species (Setosa, Versicolor, and Virginica). Each sample is described by four numerical features (sepal length, sepal width, petal length, and petal width). This dataset provides a contrasting challenge, testing the algorithms’ ability to find meaningful structure in a dense, continuous feature space, while still offering clear ground-truth classes for evaluation.

While these are classic benchmarks for flat partitioning, they serve to evaluate a hierarchical algorithm’s ability to recover known, meaningful ground-truth groupings, a common goal in exploratory data analysis where the true underlying structure may not be strictly hierarchical. The Zoo dataset, in particular, contains classes with an implicit biological hierarchy, offering a chance for qualitative assessment of the dendrogram’s structure.

## 2.3.3 Single-cell RNA sequencing data

For real-world validation, we used single-cell RNA sequencing (scRNA-seq) data from the Tabula Muris project (Schaum *et al.* 2018), a comprehensive and publicly available mouse cell atlas. This dataset comprises around 100 000 cells from 20 tissues, from which we selected the six tissues with the lowest cell counts (aorta, diaphragm, kidney, limb muscle, liver, and trachea). This choice provided a diverse set of cell types for creating heterogeneous test cases while minimizing computational costs. We then generated nine distinct, randomly combined pan-tissue datasets (1500–3500 cells each). This strategy allows us to rigorously evaluate the algorithm’s performance on biologically heterogeneous data that mimics the common challenge of integrating cells from multiple experimental batches or sources.

The ground truth for this experiment was established using the expert cell-type annotations provided by the Tabula Muris consortium. These annotations are mapped to the formal CO, a structured vocabulary of cell types. To ensure a rigorous evaluation that assesses the quality of the entire hierarchy rather than just the leaf nodes, and consistent with the methodology employed in the HiDeF study (Zheng *et al.* 2021), we expanded the annotations for each cell to include all its ancestor terms in the CO. Because the goal of hierarchical clustering is to recover nested structures at multiple resolutions, evaluating against only the finest-grained labels would fail to validate the correctness of higher-level internal nodes. This expansion ensures that a method is credited for correctly reconstructing valid lineage relationships (e.g. grouping all T-cells into a “lymphocyte” clade) even if it imperfectly resolves the finest distinctions.

## 3 Results

We empirically validated our proposed methodology, beginning with a series of ablation studies to justify its design. These studies confirmed that average linkage is the most effective final-stage algorithm (Section S1.1, available as supplementary data at *Bioinformatics Advances* online) and identified a complementary portfolio of initial views: Laplacian bisection, recursive *K*-means, and centroid linkage (Section S1.2, available as supplementary data at *Bioinformatics Advances* online). Furthermore, we validated that our blending model significantly outperforms simpler blending strategies (Section S1.3, available as supplementary data at *Bioinformatics Advances* online) and that our surrogate-assisted optimizer provides substantial performance gains over a non-adaptive search (Section S1.4, available as supplementary data at *Bioinformatics Advances* online). The detailed results of these justification experiments can be found in Section S1, available as supplementary data at *Bioinformatics Advances* online. We now present the benchmark performance of the fully integrated method.

### 3.1 Comparative benchmark

**Figure 2:**
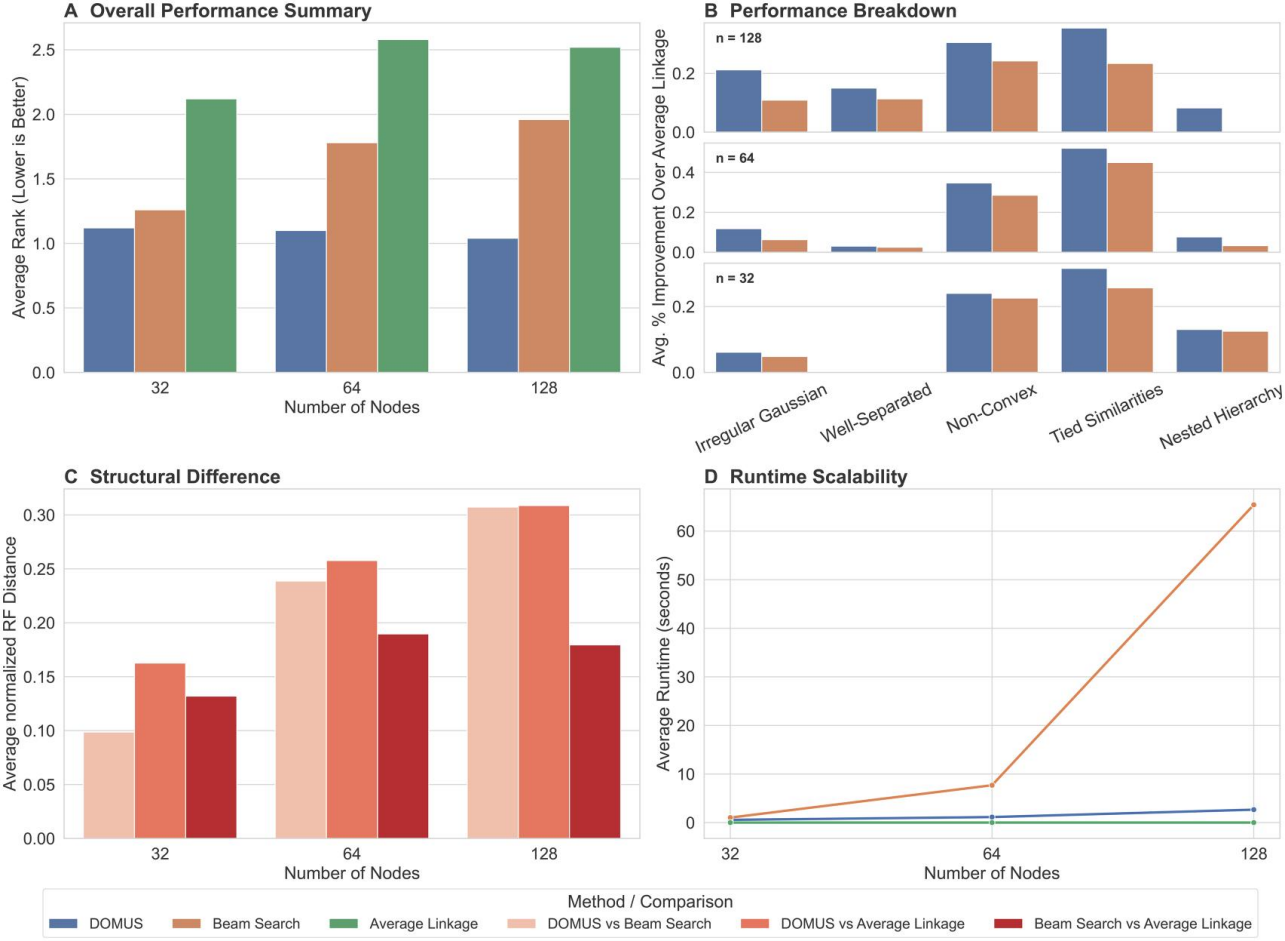
Benchmark comparison on synthetic data. The four panels compare DOMUS, average linkage, and beam search, showing: (A) average performance rank by problem size; (B) percent improvement over average linkage by data type; (C) structural dissimilarity via normalized Robinson–Foulds (RF) distance; and (D) runtime scalability.

To evaluate the overall effectiveness of our approach, we benchmarked DOMUS against average linkage and a more sophisticated beam search heuristic (average linkage with a beam width of 100 and max rank of 5, validated as the top-performing beam variant in Fig. S8, available as supplementary data at *Bioinformatics Advances* online). The results on synthetic data are shown in Fig. 2.

The four panels provide a comprehensive comparison against the baselines across performance and structural differences. Panel A shows that DOMUS consistently outperforms both baselines in terms of average rank. For each synthetic dataset, we rank the methods based on the Dasgupta cost of their output dendrograms, where a lower cost yields a better (smaller) rank. The reported value is the average of these ranks over all datasets for a given problem size. The advantage of DOMUS over beam search becomes more pronounced as the problem size increases, achieving a nearly perfect rank of 1 for n=128. Panel B provides deeper insight by breaking down the percent improvement over the average linkage baseline by data type. It shows that while DOMUS consistently outperforms the other methods across all data generator types, its advantage is most significant on more complex data.

Panel C, showing the average normalized Robinson–Foulds (RF) distance, reveals that DOMUS produces dendrograms that are structurally different from those produced by the two linkage-based baselines. The RF distance measures the dissimilarity between two dendrograms based on the number of clusters (bipartitions) that are present in one tree but not the other, normalized to a scale of 0 (identical) to 1 (maximally different). The distance between DOMUS and the baselines grows with problem size, reaching a value of around 0.3 for n=128, while beam search and average linkage produce trees that are considerably more similar (0.18). This suggests that DOMUS explores a different, more effective part of the solution space.

The runtime of DOMUS grows moderately, demonstrating a practical quadratic scaling that is superior to the beam search heuristic’s cubic complexity (Panel D). A full, detailed runtime analysis is provided in Section S3 and Fig. S10, available as supplementary data at *Bioinformatics Advances* online.

To further contextualize these results, we also evaluated a spectral clustering algorithm utilizing the Laplacian Fiedler vector and the state-of-the-art, graph-based community detection algorithm HiDeF (Zheng *et al.* 2021). As detailed in Fig. S11, available as supplementary data at *Bioinformatics Advances* online, these additional methods consistently performed worse than the standard average linkage baseline on this benchmark.

To provide a clear, mechanistic test of our core hypothesis that reshaping the similarity space can guide a greedy heuristic, we conducted a benchmark on a specialized dataset known to deceive average linkage. As described in Section 4, this dataset is constructed with two competing structures: a set of small, dense “decoy” cliques and a set of larger, globally “optimal” cliques. The higher internal similarity of the decoy cliques is designed to trap average linkage into forming suboptimal clusters. The results of this targeted experiment are shown in Fig. S12, available as supplementary data at *Bioinformatics Advances* online, which plots the percent improvement over the standard average linkage baseline for different problem sizes (controlled by the parameter *k*). The performance of the theoretically optimal clustering is shown as a dashed red line. The results provide a clear validation of our approach: For all tested problem sizes, DOMUS successfully avoids the local optima presented by the decoy cliques and achieves a score improvement that matches the optimal possible improvement. Conversely, the beam search heuristic shows zero percent improvement, meaning its performance is identical to that of the standard average linkage algorithm. This result highlights the core difference in strategy: beam search attempts to overcome the heuristic’s local optima by exploring more paths, whereas DOMUS succeeds by fundamentally reshaping the similarity landscape so that the heuristic is no longer deceived by the local optima in the first place.

#### 3.1.1 Benchmark on classic datasets

We further evaluated DOMUS on two real-world datasets, the Zoo dataset (n=101) and the Iris dataset (n=150). For both experiments, the evaluation is based on how well the resulting hierarchy conforms to the known ground-truth classes.

To generate the initial similarity matrix *W* for each dataset, we used a method appropriate for its feature types. For the Zoo dataset, which consists of primarily Boolean features, we used Hamming distance ($S=1-D_{\mathrm{hamming}}$). This provides a simple, parameter-free method for generating a base similarity matrix, though it treats the single numeric “legs” feature as categorical. For the continuous numerical features of the Iris dataset, we first calculated the pairwise Euclidean distance and then converted it to a similarity matrix using a Gaussian kernel.

In both cases, each algorithm takes the respective *W* matrix as its input to generate a hierarchy, while our method’s *K*-means view operates directly on the original feature matrix, its natural input format. The cost of the final dendrogram produced by each method is calculated against a ground-truth similarity matrix, $W_{\mathrm{true}}$, where similarity is 1 for items within the same class and 0 otherwise. This provides a standard, objective method for comparing how well each dendrogram recovers the primary class structure.

**Figure 3:**
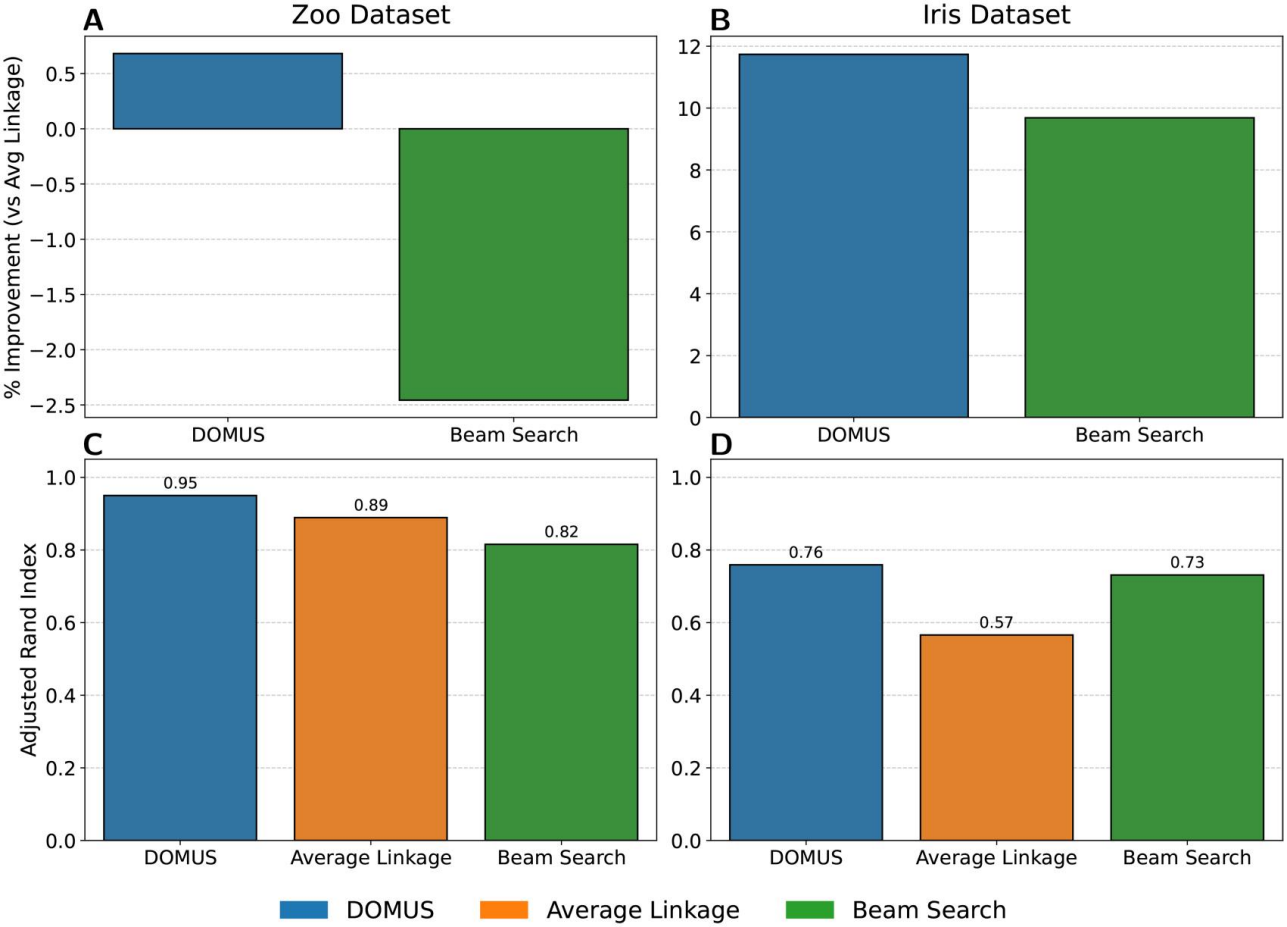
Benchmark comparison on the Zoo (left panels: A and C) and Iris (right panels: B and D) datasets. The top panels (A and B) show the percent improvement in Dasgupta’s cost over the average linkage baseline (higher is better). The bottom panels (C and D) show the Adjusted Rand Index (ARI) for flat partitions obtained by cutting the dendrogram at the ground-truth number of clusters (higher is better).

The results for both benchmarks are shown in Fig. 3. Unlike the synthetic experiments, where we evaluated the cost on the input similarity matrix, here we calculate the cost against the ground-truth class labels to objectively measure structural recovery. The top panels display the improvement in this ground-truth Dasgupta cost relative to the average linkage baseline. On the Zoo dataset, DOMUS achieved a quantitative improvement of ∼0.75% (panel A). While numerically modest, this reduction corresponds to the correction of specific topological errors, as detailed in the case study below. In contrast, the beam search heuristic performed significantly worse, degrading performance by nearly 2.5%. On the Iris dataset (panel B), the importance of regularization became evident. DOMUS achieved a substantial 12% improvement, outperforming beam search (10%). Notably, the non-regularized “optimal” variant—which minimizes the cost on the input matrix directly—yielded only a 5% gain on this metric (data not shown). This discrepancy highlights that while the “optimal” variant serves as a theoretical tool for minimizing the objective, it is prone to overfitting noise in complex data. The “robust” variant, by optimizing for stability across bootstraps, prevents this overfitting and is therefore the recommended approach for practical applications. To verify that these structural improvements translate into practical utility, we evaluated the adjusted rand index (ARI) of the flat partitions obtained by cutting the dendrograms at the ground-truth number of classes (k=7 for Zoo, k=3 for Iris). The results, displayed in the bottom panels of Fig. 3, strongly favor DOMUS. On the Zoo dataset (panel C), it achieves an ARI of 0.95, a near-perfect recovery of ground truth classes, and a significant improvement over the average linkage baseline (0.89) and beam search (0.82). On the Iris dataset (panel D), the value of optimization becomes even clearer. Both optimization approaches significantly outperformed the greedy baseline (ARI 0.57): beam search achieved a strong ARI of 0.73, while DOMUS reached 0.76. This relative improvement of over 30% in the ARI score on the Iris dataset confirms that optimizing the global hierarchical objective directly correlates with superior flat partitions, with DOMUS providing the most robust performance across diverse data topologies.

Finally, we analyzed the structural similarity of the resulting dendrograms using the normalized RF distance. This analysis revealed that DOMUS produced hierarchies that were structurally distinct from the average linkage baseline (RF distance 0.5–0.6 on Zoo, 0.3–0.4 on Iris). Furthermore, our robust and optimal variants found different structures from one another (RF distance around 0.45 on Zoo and 0.25 on Iris). In comparison, the dendrogram produced by beam search was considerably more similar to the baseline structure (0.33 on Zoo, 0.24 on Iris). This reinforces that DOMUS, in both its forms, is not merely making small adjustments but is finding fundamentally different and more effective hierarchical structures in the data.

A qualitative analysis of the Zoo dataset provides a tangible example of these structural improvements. In a targeted case study on the well-known biological subclade of “flightless birds” (kiwi, ostrich, penguin, and rhea), DOMUS perfectly recovered the group with an F1-score of 1.00 (Fig. S13, available as supplementary data at *Bioinformatics Advances* online). In contrast, the baseline average linkage method failed to identify this structure, achieving an F1-score of only 0.44, as its best-matching cluster was both impure and incomplete. This case study provides a clear example of how our method’s quantitative cost reduction corresponds to a qualitatively superior and more biologically meaningful hierarchy.

To ensure our method’s performance gains did not come at the cost of statistical robustness, we performed a Jaccard-based bootstrap analysis to assess cluster stability. The analysis confirmed that the clusters produced by DOMUS are as stable as those from the baseline average linkage, with the most stable clusters approaching the theoretical maximum stability. This indicates that our framework finds superior hierarchical structures without sacrificing reproducibility. The full details of this analysis can be found in Section S5, available as supplementary data at *Bioinformatics Advances* online.

As with the synthetic data, we also compared DOMUS against the spectral clustering and HiDeF baselines on the classic datasets. The results, shown in Fig. S14, available as supplementary data at *Bioinformatics Advances* online, demonstrate our method’s superior performance in this supervised label-recovery setting.

#### 3.1.2 Application to single-cell RNA sequencing data

To validate our method’s performance on a complex, real-world task, we evaluated its ability to recover the known biological hierarchy of cell types from single-cell RNA sequencing (scRNA-seq) data. For this benchmark, we adopted the experimental design from the paper that introduced HiDeF (Zheng *et al.* 2021) to ensure a direct and rigorous comparison.

Following the procedure established in the HiDeF paper, we used a standard preprocessing pipeline where counts were log-normalized, the top 2000 highly variable genes were selected, and the dimensionality was reduced to 30 principal components using PCA. The primary similarity matrix, *W*, which served as the input for all methods, was a Shared Nearest Neighbor (SNN) graph constructed from this PCA embedding. This choice is critical, as the SNN graph is the direct input for HiDeF and represents a state-of-the-art technique for capturing the local neighborhood structure in scRNA-seq data. The communities produced by each method were then ranked to assess performance as a function of model complexity. HiDeF communities were ranked by their internal “persistence” score, as described in the original paper. For DOMUS, each community (i.e. each branch in the dendrogram) was ranked using a one-way Mann–Whitney *U* test—a statistical approach also utilized in the HiDeF paper. This test assesses whether the distances between cells in the two sibling communities being merged are significantly greater than the distances between cells within each of those communities. For this statistical test, we used distances derived from a Pearson correlation-based similarity matrix, as it provides a robust, global measure of a cluster’s coherence. The communities were then sorted by the resulting *P*-value. Performance was measured using the average F1 score, which compares the top *N*-ranked communities from each method against the full set of ground-truth cell types.

**Figure 4:**
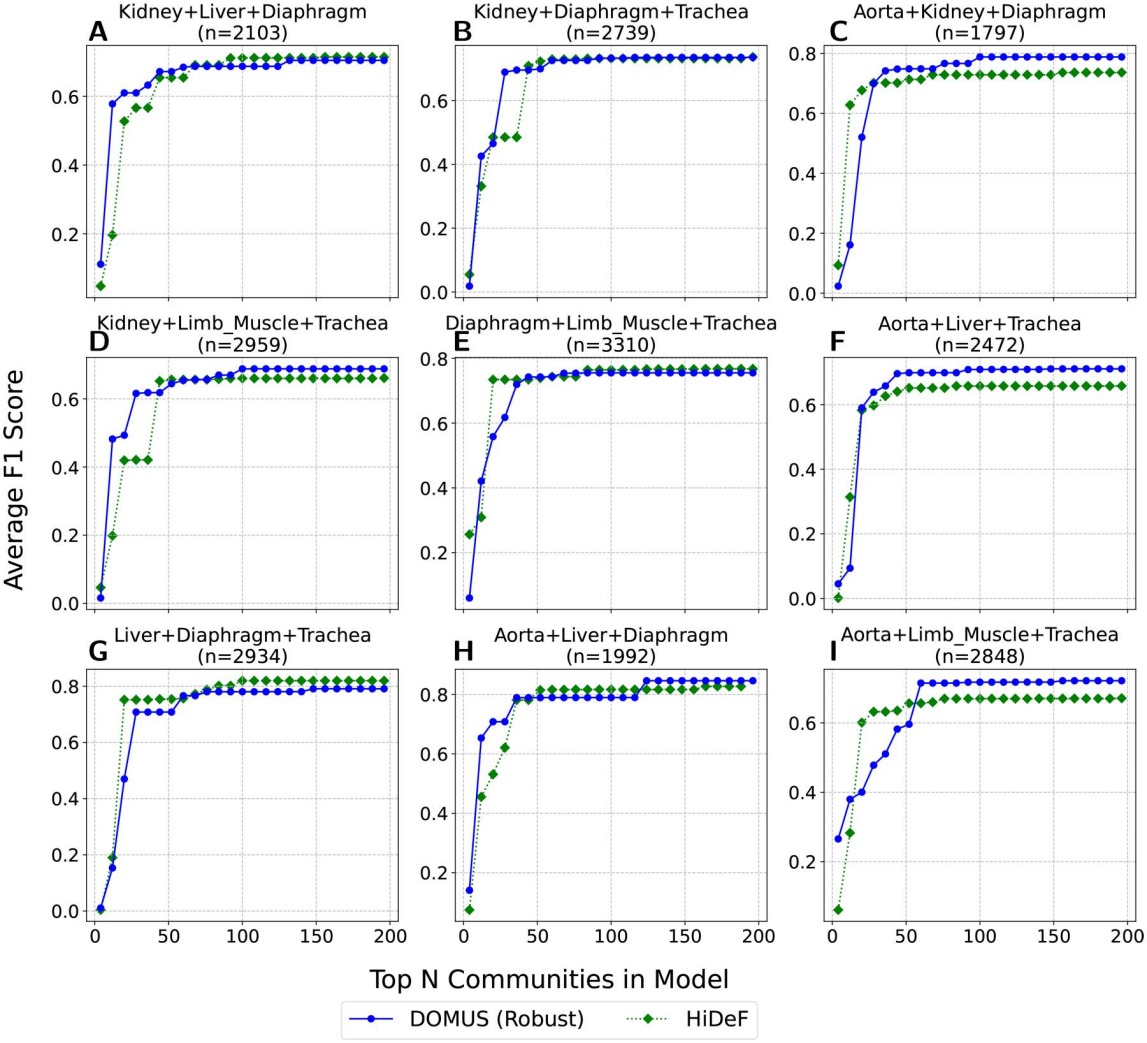
Performance comparison on scRNA-seq data. The average F1 score for cell type hierarchy recovery is plotted against the number of top-ranked communities for DOMUS and the HiDeF baseline. (A-I) The experiment was run on nine pan-tissue combinations of the six smallest datasets from the Tabula Muris project.

The results, summarized across the nine pan-tissue datasets in Fig. 4, show that our method’s performance is highly competitive with the state-of-the-art HiDeF algorithm. While the interpretation is complex due to the intersecting performance curves, DOMUS often achieves a higher average F1 score across the broader range of top *N* communities. We observe that HiDeF frequently performs strongly for the highest-ranked communities, consistent with its design to identify highly persistent local modules.

However, this comparison must be interpreted in light of the problem’s underlying structure. The ground truth Cell Ontology (CO) is a directed acyclic graph (DAG) where cell types can have multiple parents—a structure that HiDeF’s overlapping communities can natively represent, whereas DOMUS is constrained to produce a strict binary tree. The fact that DOMUS achieves competitive performance despite this structural constraint suggests that it successfully recovers the primary hierarchical relationships that define the cellular taxonomy.

**Figure 5:**
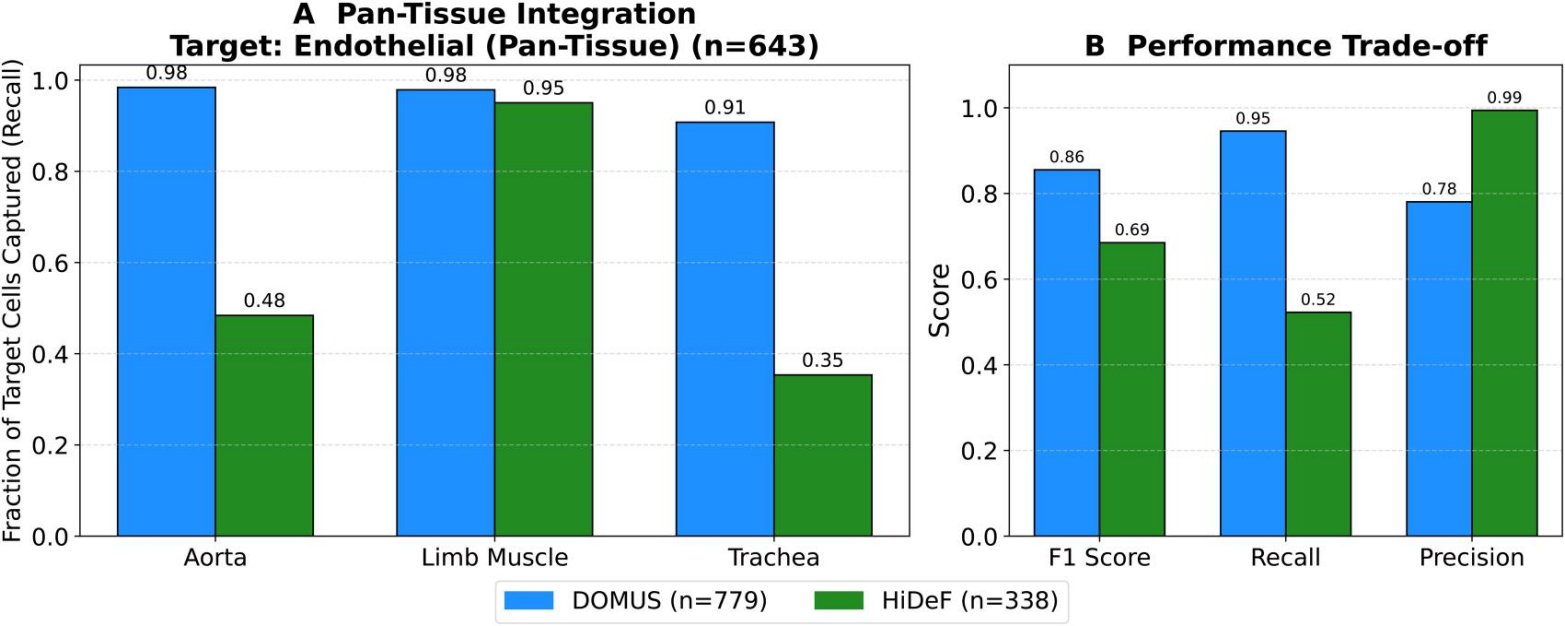
Case study on pan-tissue lineage recovery. Comparison of DOMUS and HiDeF on the recovery of the endothelial lineage across three distinct tissues (aorta, limb muscle, trachea). (A) Pan-Tissue Integration: DOMUS achieves high recall across all three tissues (>0.91), effectively bridging tissue-specific transcriptomic differences to identify a unified “Pan-Tissue endothelial” clade. In contrast, HiDeF is biased toward the Limb Muscle, capturing <50% of the endothelial cells from the aorta and trachea. (B) Performance Tradeoff: While HiDeF achieves near-perfect precision (0.99) by identifying a highly specific local community (n=338), it suffers from low overall recall (0.52). DOMUS achieves a significantly higher F1 score (0.86 vs 0.69) by prioritizing overall recall (0.95), successfully recovering the complete cellular lineage (n=779) at the cost of modest precision (0.78).

This structural tradeoff is clearly illustrated in our Pan-Tissue Endothelial case study (Fig. 5). To investigate the differences, we focused on the endothelial lineage, a ubiquitous cell type present across all vascularized tissues that should theoretically form a cohesive global clade. We identified the single cluster from each method that maximized the F1 recovery of the ground-truth endothelial lineage in a representative dataset combining distinct anatomical sites: aorta, limb muscle, and trachea. HiDeF identified a highly precise community (P=0.99); however, inspection revealed that this cluster (n=338) was dominated by a single tissue (limb muscle), capturing <50% of the endothelial cells from the aorta and trachea. In contrast, DOMUS, constrained to find a global hierarchy, successfully unified these disparate tissues into a single “Pan-Tissue Endothelial” clade (n=779) with consistent high recall across all three tissues (>91% per tissue, 95% overall). This global recovery yielded a significantly higher F1 score for DOMUS (0.86) compared to HiDeF (0.69). These findings highlight the complementary nature of the two approaches: while HiDef excels at isolating highly specific, local subsets, DOMUS provides the global hierarchical scaffold necessary to reconstruct the broader backbone that connects distinct functional units across the atlas.

### 3.2 Analysis of the adaptive blending mechanism

**Figure 6:**
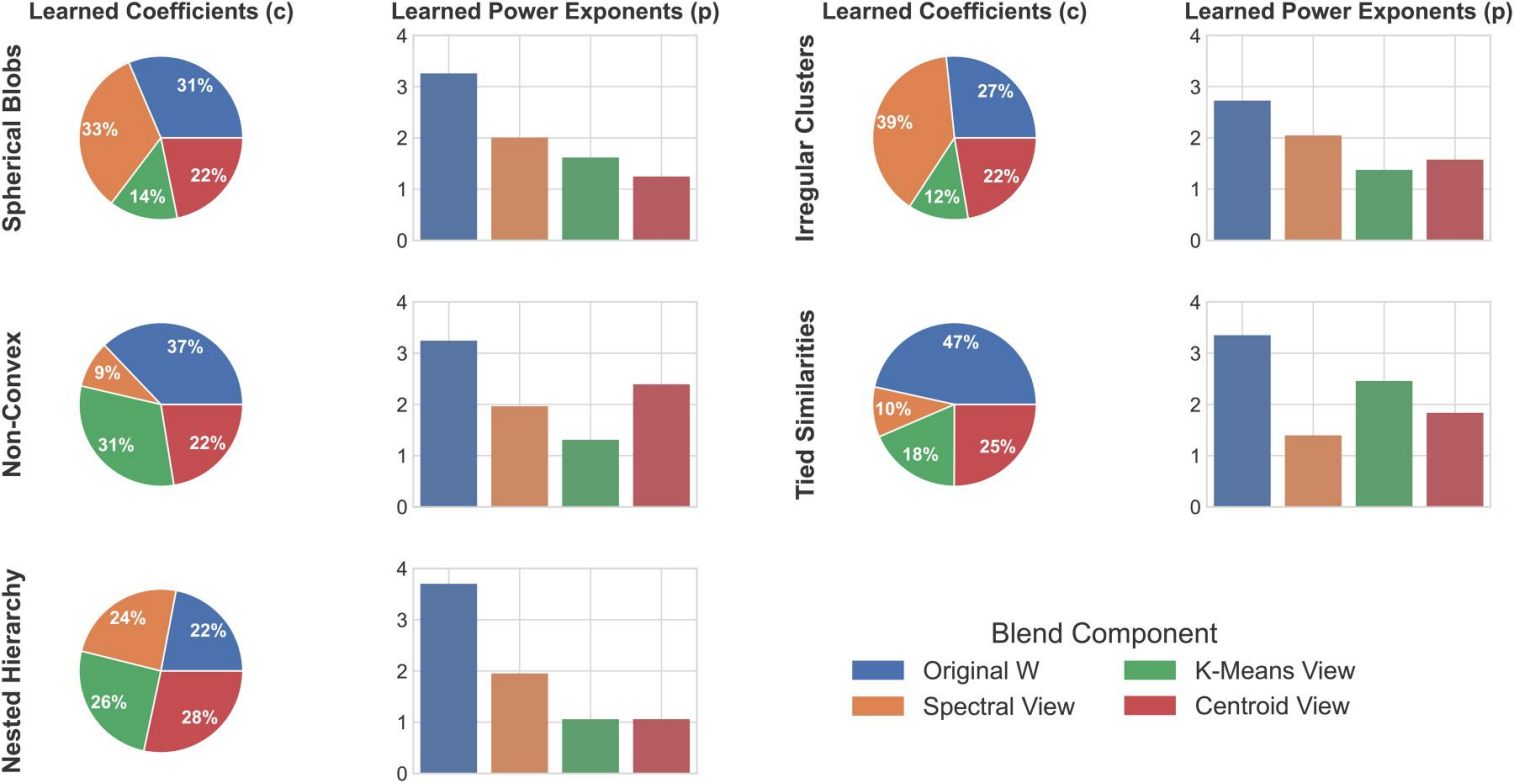
Mechanistic analysis of the learned blending strategy. This figure visualizes the optimizer’s learned parameters for the “Flexible Interaction” model across five distinct data archetypes. Each row corresponds to one archetype. (Left) The pie charts show the average learned coefficients ($c_{i}$), representing the proportional contribution of the original similarity matrix (*W*) and the three derived views (spectral, *K*-means, centroid) to the final blended matrix. (Right) The bar charts show the average learned power exponents (*p*), which control the non-linear rescaling of each component’s similarities. Collectively, the figure illustrates how the method adaptively learns a different blending “recipe” to best suit the intrinsic structure of each dataset.

To better understand the inner workings of DOMUS, we analyzed its adaptive blending strategy on five canonical data archetypes (see Fig. 6 for the full results visualization). The results suggest that the optimizer learns a two-part strategy: it aggressively sharpens the original similarity matrix to amplify trustworthy local signals and learns a data-dependent “recipe” to blend in corrective information from the most appropriate derived views. This analysis demonstrates the critical role of the flexible power exponents in our model, which enable a universal strategy of signal sharpening, and the coefficient weights, which tune the blend to the unique topology of a given dataset.

This adaptive learning is evident across the dataset types. For example, on discrete data with tied similarities, the optimizer learns to trust the sharpened original matrix almost exclusively, assigning it the highest coefficient weight by a large margin. For irregular clusters, the spectral view is assigned the highest coefficient, highlighting its role in finding a global structure. For nested hierarchies, the optimizer learns a balanced portfolio where all components contribute almost equally. The results for non-convex show a different strategy. In this case, the optimizer assigned a low weight to the spectral view, instead favoring a combination of the heavily sharpened original matrix and a high weight on the divisive *K*-means view. This learned parameterization demonstrates the model’s ability to find effective, and sometimes non-intuitive, recipes for complex topologies. Overall, this analysis confirms that our method’s effectiveness stems from its ability to diagnose a dataset’s intrinsic structure and learn an optimal, adaptive blend of the most appropriate analytical views.

## 4 Conclusion

We introduced a novel framework for hierarchical clustering, DOMUS, that aims to minimize Dasgupta’s cost. Rather than relying on greedy heuristics that are prone to finding suboptimal solutions, DOMUS first generates a portfolio of diverse structural views of the data, which are then blended—using a surrogate-assisted algorithm—into a single, optimized similarity matrix designed to steer a simple final-stage heuristic toward a superior solution.

Our empirical evaluation confirmed the utility of this approach. On a wide range of synthetic benchmarks, DOMUS consistently outperformed both standard average linkage and a more sophisticated beam search heuristic and successfully identified the optimal structure in a targeted experiment designed to trap greedy algorithms. The method’s advantage was also evident on classic real-world datasets (Zoo and Iris), where DOMUS found dendrograms that were not only lower cost but also structurally distinct from those produced by the baselines. Finally, we validated DOMUS on the complex and challenging task of cell type hierarchy recovery from single-cell RNA sequencing data.

Despite these strong results, our framework has limitations that suggest clear directions for future work. The primary constraint is runtime scalability; while its overall complexity will eventually be dominated by the super-quadratic spectral view generation step, the iterative optimization loop remains a significant cost. To address the computational bottlenecks associated with both the initial hierarchy generation and the iterative optimization loop, we propose a scalable two-stage inference strategy: the optimal blending parameters can be learned on a representative subsample of the data and then applied to the complete dataset. Implementing this subsampling approach will be a primary focus of future work, as it is the key to scaling DOMUS to evaluate larger top *N* communities and massive biological networks, such as protein–protein interaction (PPI) networks and diverse omics data. Furthermore, for atlas-level data where the spectral computation itself is prohibitive, the framework’s modularity allows this view to be replaced by a scalable alternative or excluded from the portfolio entirely, enabling the optimization of a lightweight blend of highly scalable derivation methods. Beyond these inference strategies, the optimization overhead could be further reduced by using meta-learning to create a library that maps features of similarity matrices to promising blending parameters, providing better initializations and reducing the search budget. Apart from performance considerations, the learned parameters themselves offer a compelling avenue for research. Interpreting the optimal weights and exponents could provide a diagnostic signature for a dataset, revealing whether its underlying structure is more graph-like (e.g. high weight on the Laplacian view) or composed of dense clusters (e.g. high weight on the *K*-means view). Finally, the modularity of the framework itself invites extension. By swapping its core components—the initial derivers, the blending model, or the final-stage algorithm—the method could be adapted to optimize different cost functions or to produce more complex structures like non-binary or overlapping hierarchies, greatly broadening its applicability.

## Supplementary Material

vbag107_Supplementary_Data

## Data Availability

The Zoo and Iris datasets are publicly available from the UCI Machine Learning Repository, at https://archive.ics.uci.edu. The Tabula Muris single-cell RNA sequencing data is available at https://tabula-muris.sf.czbiohub.org/data. The synthetic data generation scripts are available alongside the code in the DOMUS GitHub repository, at https://github.com/GalGilad/DOMUS.

## Code availability

The code for DOMUS is publicly available on GitHub under: https://github.com/GalGilad/DOMUS.
