# Supplementary material for: An optimization framework for hierarchical clustering
Source: Bioinform Adv. 2026 Apr 13;6(1):vbag107. doi: 10.1093/bioadv/vbag107 (PMC13128330; doi:10.1093/bioadv/vbag107)
Supplement: vbag107_Supplementary_Data [file vbag107_supplementary_data.pdf]

Supplementary Material for: An optimization framework for hierarchical clustering

Gal Gilad* Roded Sharan*

*School of Computer Science and AI, Tel Aviv University, Tel Aviv 69978, Israel.

Supplementary Material

S1 Justification of Methodological Design Choices

S1.1 Justification of Final Linkage Algorithm

The final step of our pipeline takes a blended similarity matrix S_{blend} and produces a dendrogram. We conducted an experiment comparing the performance of all candidate algorithms available to our framework for generating initial views (referred to here as ”derivers”) when used as standalone clustering methods on the original similarity matrix W. The results, summarized over 50 datasets for each problem size (see Figure S1), showed that Agglomerative Average Linkage was significantly and consistently better than all other methods, achieving the lowest average rank by a clear margin. These results strongly suggest that, among the tested algorithms, the merging strategy of Average Linkage serves as the best heuristic proxy for minimizing Dasgupta's Cost from a similarity matrix. We acknowledge that fixing Average Linkage as the final-stage algorithm is a key design choice; the optimization is therefore geared toward producing a similarity matrix from which this specific heuristic can construct a low-cost dendrogram. Based on these results, we selected Average Linkage as the fixed final-stage algorithm for all subsequent experiments.

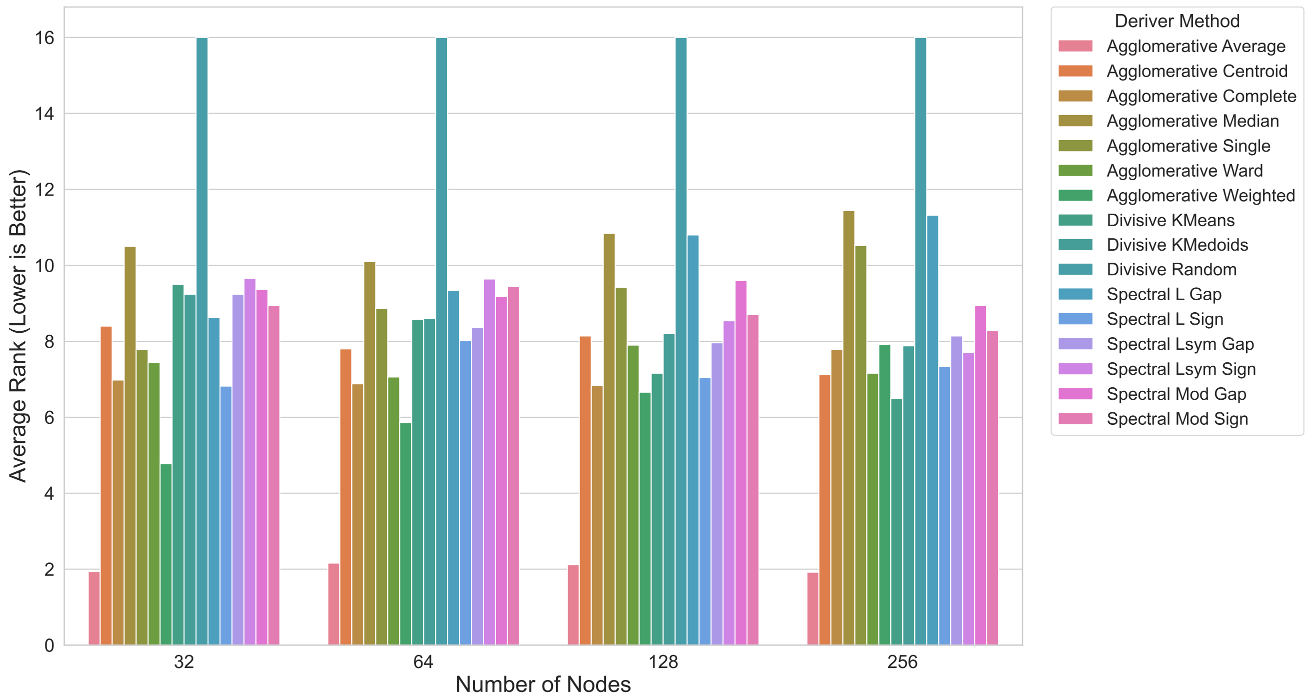

Figure S1: Performance comparison of all standalone deriver algorithms. Average Linkage consistently achieves the best (lowest) average rank across different problem sizes.

S1.2 Selection of Initial Views

The core of our approach is to blend multiple diverse ”views” of the data, making the selection of these initial views critical. The goal is not simply to find methods that are individually strong on average, but rather to assemble a complementary portfolio of views. An ideal set is one where, for any given problem, at least one of the views is likely to provide a high-quality perspective on the data's structure. To identify such a set, we first evaluated each individual deriver based on its **performance potential**. This potential is measured by its ability to

improve upon the original similarity matrix W when used in a simple blend, which is then evaluated using the final-stage Average Linkage algorithm, as justified in the previous section. Specifically, for each deriver, we create a derived matrix S and find the optimal cost from a linear blend, $\min_{\alpha \in [0,1]} \mathrm{Cost}(\alpha W + (1-\alpha) S)$. This cost serves as a more accurate proxy for a deriver's utility within our specific blending framework than its standalone performance.

The results of this analysis are visualized in Figure S2. The performance bar chart shows that spectral and divisive methods generally have higher potential than agglomerative ones. Specifically, Laplacian Bisection (By Gap) and Bisecting K-Means emerged as the top performers. Unsurprisingly, most linkage-based methods ranked the lowest, as their underlying logic of assessing local connections is too similar to our final-stage Average Linkage algorithm, thus offering redundant information. The notable exception was Centroid Linkage, which ranked among the better methods. We speculate its success stems from the orthogonal perspective it provides - instead of local connections, it compares clusters based on their global similarity profiles, offering the unique structural information our blender can exploit.

With this measure of potential for each deriver, we then searched for the optimal complementary set. We scored every possible subset of size $k = 3$ by evaluating its "best-member" performance—that is, for each dataset, we considered only the performance of the single best view within that subset, and then averaged this score across all datasets. This approach directly implements our goal of finding a complementary portfolio and identified the set of {**Laplacian Bisection (By Gap)**, **Recursive K-Means**, **Centroid Linkage**} as the lowest-cost triplet. While we also computed the diversity between methods (see Figure S2, right panel), we confirmed that these three chosen methods were sufficiently diverse and proceeded with this empirically best-performing set for all subsequent experiments.

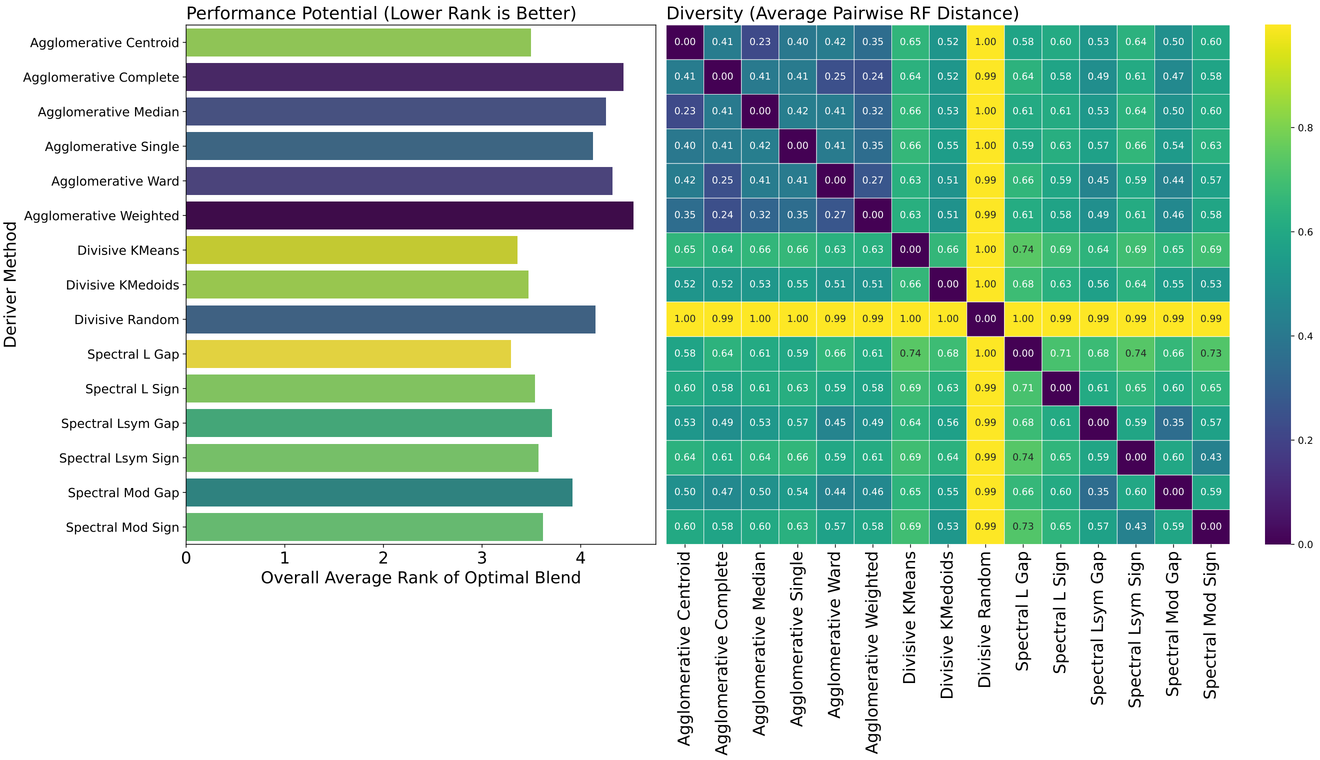

Figure S2: Analysis of deriver performance potential and diversity. Left: Average rank of each method's potential cost. Right: Heatmap of average pairwise RF distance, where lighter colors indicate greater structural diversity.

The three selected methods, which are used as the initial views for the blending pipeline,

are described in detail below:

1. **Recursive Laplacian Bisection (By Gap):** This method interprets the similarity matrix W as a graph's adjacency matrix. While standard spectral clustering into k partitions typically utilizes k eigenvectors, we employ recursive spectral bisection. At each step, we treat the splitting problem as a graph bipartition minimizing the Ratio Cut, for which the single Fiedler vector is the theoretically appropriate relaxation. It computes the Fiedler vector—the eigenvector corresponding to the second-smallest eigenvalue of the graph Laplacian. The items are then partitioned by sorting the Fiedler vector's components and splitting at the largest gap between consecutive values. This process is applied recursively to form a full binary dendrogram, leveraging global graph structure for partitioning.

2. **Recursive K-Means:** This is a top-down divisive approach. It begins with all data points in a single cluster, which is then split into two sub-clusters using the K-Means algorithm with $k = 2$. This bisection process is applied recursively to the resulting sub-clusters until each cluster contains only a single data point. This method relies on identifying dense, spherical sub-regions within the data.

3. **Centroid Linkage:** This is a bottom-up agglomerative method that starts with each data point in its own cluster. In each step, it merges the two clusters whose centroids (mean vectors) are closest. The distance between the centroids of any two clusters defines their dissimilarity. This process continues until all points are merged into a single cluster, forming the dendrogram.

S1.3 Validation of the Blending Strategy

Having selected the initial views, we next validate the blending model itself. We conducted two experiments: one to justify our choice of the more complex Flexible Interaction model and another to confirm the synergistic benefit of using multiple views.

We consider two simple blending models:

1. **No Interaction Model:** This model includes power parameters for each derived matrix but omits the interaction term with W.

$$S_{\text{blend}} = c_0 W + \sum_{i=1}^{k} c_i S_i^{p_{si}} \tag{S1}$$

2. **Linear Model:** This is the simplest model, representing a basic weighted average of the matrices without any power parameters or interactions.

$$S_{\text{blend}} = c_0 W + \sum_{i=1}^{k} c_i S_i \tag{S2}$$

All coefficients and power exponents are subject to the same constraints as in the Flexible Interaction model.

As shown in Supplementary Figure S3, the Flexible Interaction model completely dominates the other two approaches, achieving an average rank near 1.0 across all problem sizes. This result strongly confirms that modeling the complex, non-linear interactions between the original data and the derived views is crucial for achieving better performance.

Second, we validated that combining our three chosen views is more effective than using smaller subsets. We compared the performance of the full three-view model against all possible single-view and two-view combinations. The results in Supplementary Figure S4 demonstrate

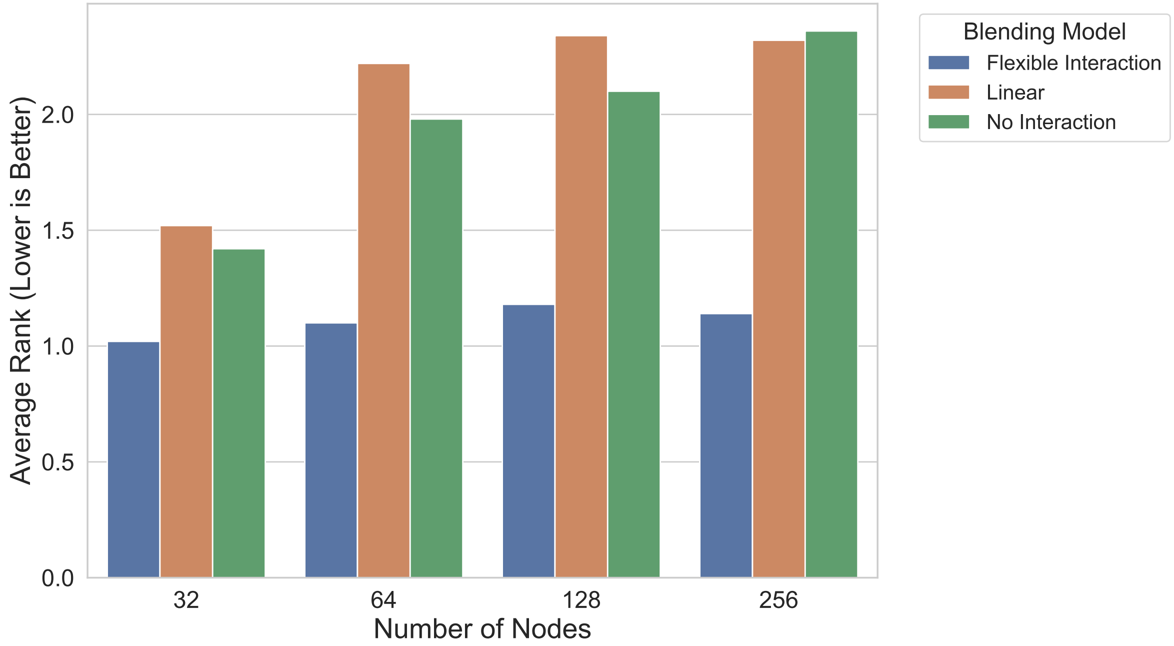

Figure S3: Comparison of the three blending models. The Flexible Interaction model consistently and significantly outperforms the simpler Linear and No Interaction models.

clear synergy. The model using all three chosen views consistently outperforms all smaller subsets. This confirms that each view contributes unique structural information that is effectively integrated by the blending optimizer.

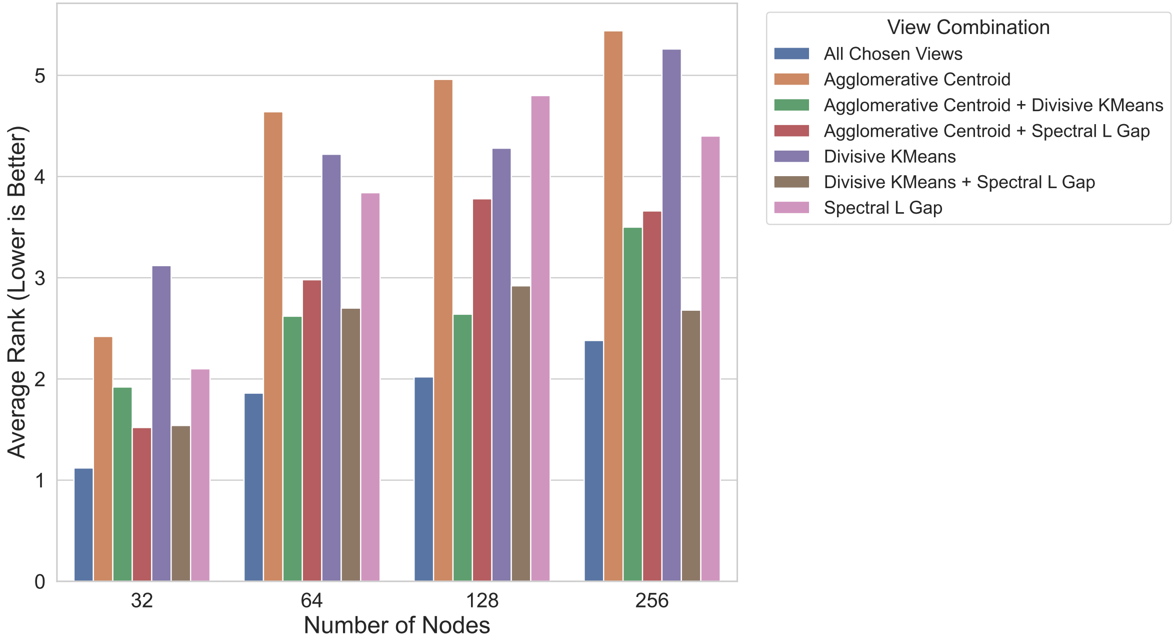

Figure S4: Synergy analysis of the chosen view combinations. Using all three views consistently achieves a better (lower) average rank than using any single-view or two-view subset.

S1.4 Analysis of the Optimization Algorithm

The surrogate-assisted optimization algorithm (illustrated in Supplementary Figure S5) balances broad exploration with focused exploitation through three main phases:

1. **Initial Exploration via LHS Warm-up:** The process begins by generating an initial population of candidate parameter vectors using Latin Hypercube Sampling (LHS). This ensures a wide, quasi-random coverage of the search space. Each of these candidates is evaluated using the full, more expensive objective function to provide an initial map of the performance landscape.

2. **Surrogate Model Training:** Immediately following the warm-up, a fast surrogate model (a balanced Logistic Regression classifier) is trained a single time on the initial population. The model learns to predict whether a given parameter vector is likely to belong to the top-performing half of the population. This creates a computationally cheap proxy for the true objective function, which will be used to guide subsequent exploration. We note that the simple Logistic Regression classifier was chosen as the surrogate model to mitigate the risk of overfitting in this low-data regime.

3. **Iterative Refinement:** The algorithm then proceeds in a series of rounds, where the population of candidates is progressively refined. To ensure a predictable and efficient search, the total post-warm-up computational budget is deterministically partitioned and scheduled before the refinement loop begins. Each round balances exploitation and exploration:

   - **Exploitation:** The top portion of the current population (the "elites") are selected. Each elite candidate undergoes a local hill-climbing search to refine its parameters, governed by a pre-allocated exploitation budget that ensures resources are spent in every round.
   - **Exploration:** The pre-trained surrogate model is used to screen a large number of newly generated LHS candidates. It efficiently predicts their performance, and a number of the most promising new candidates are selected for full evaluation. To prioritize broad exploration early on, the budget for these injections is front-loaded using a weighted decay schedule, injecting more candidates in the initial rounds and tapering off toward the end.
   - **Population Update:** The next generation is formed by combining the refined elites with the newly injected candidates, focusing the computational budget on increasingly promising areas of the parameter space.

This cycle repeats for a pre-calculated number of rounds to ensure the entire budget is used, effectively navigating the trade-off between deepening the search around known good solutions and discovering entirely new ones.

To analyze this algorithm and justify its design, we performed two experiments: an ablation study to validate its effectiveness over a simpler baseline and a budget analysis to understand its scaling properties.

First, we compared our surrogate-assisted approach against a simpler baseline that uses its entire computational budget on the initial Latin Hypercube Sampling (LHS) with no subsequent refinement. The results in Supplementary Figure S6 show a clear and significant benefit to the surrogate-assisted approach across the range of tested problem sizes. Importantly, the bottom panel shows that this performance gain comes at virtually no cost in runtime, making it a highly efficient strategy.

Second, we examined the effect of the total iteration budget on the final solution quality. Figure S7 shows that performance consistently improves as the budget increases from 50 to

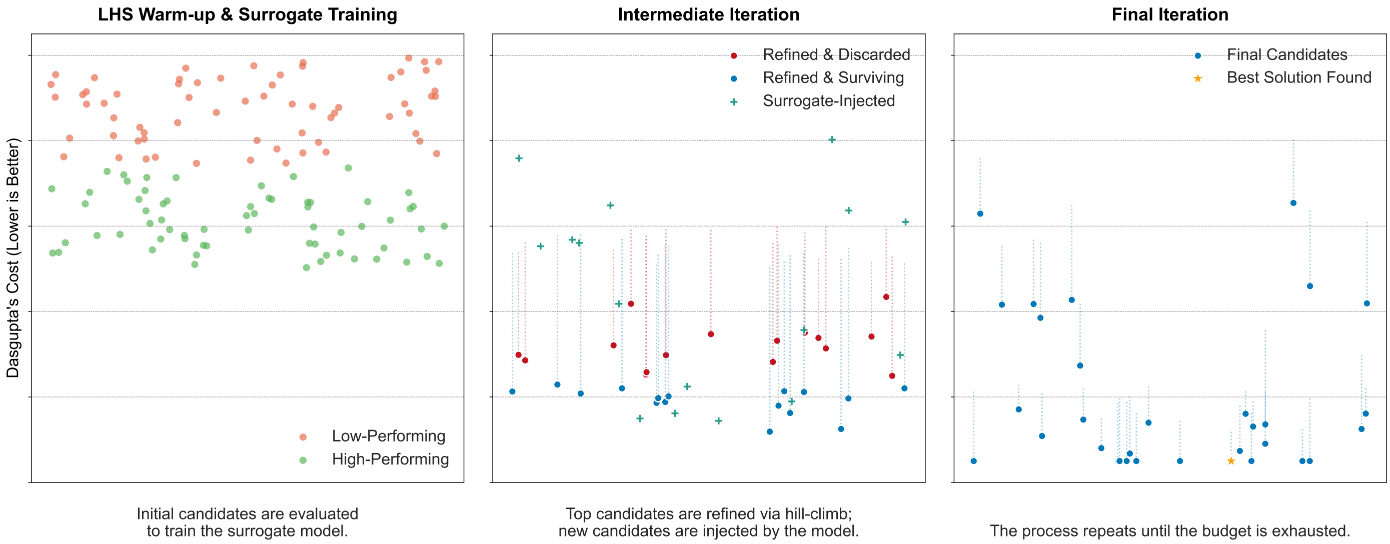

Figure S5: **The surrogate-assisted optimization process.** The three panels illustrate the algorithm's progression. **(Left)** An initial population is generated via Latin Hypercube Sampling (LHS) and evaluated to train the surrogate model. **(Middle)** The algorithm iteratively refines top candidates (hill-climbing) and uses the surrogate model to inject new, promising candidates. **(Right)** The process continues until the budget is exhausted, identifying the final population and the single best solution found.

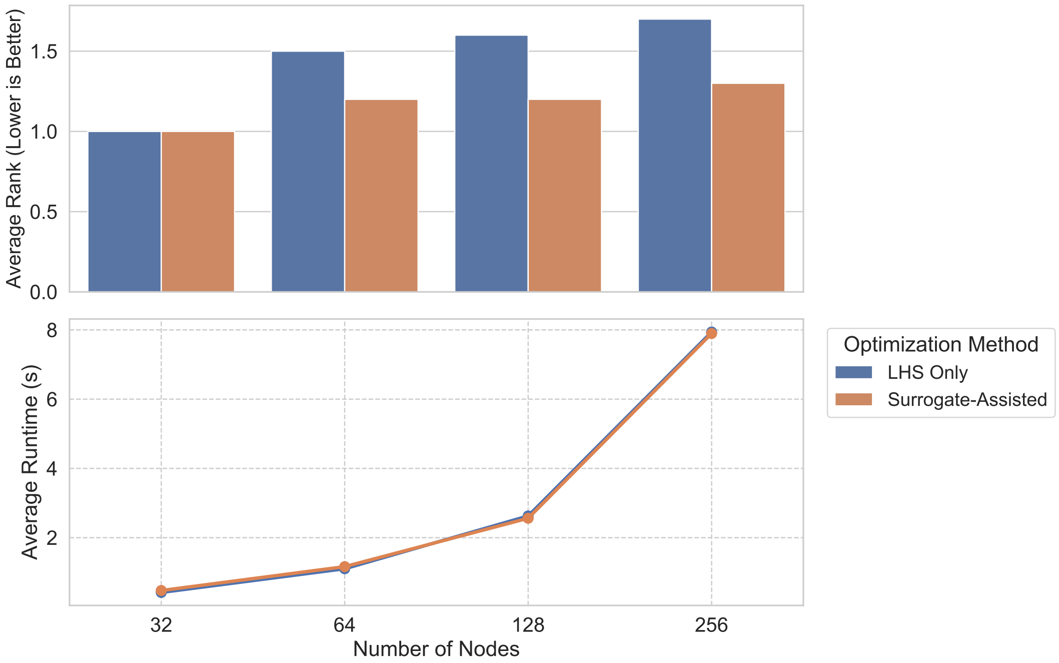

Figure S6: Ablation study of the optimization algorithm. Top: Performance rank of the full surrogate-assisted method vs. LHS-only initialization. Bottom: Corresponding average runtime.

1000 iterations, with no clear evidence of plateauing for larger problem sizes. The fact that performance gains do not saturate suggests that the parameter space is highly complex and that for challenging problems, the algorithm could benefit from an even larger search budget, highlighting a potential area for future work on more advanced search heuristics. The bottom panel confirms that the runtime scales linearly with the iteration budget, as expected. A full

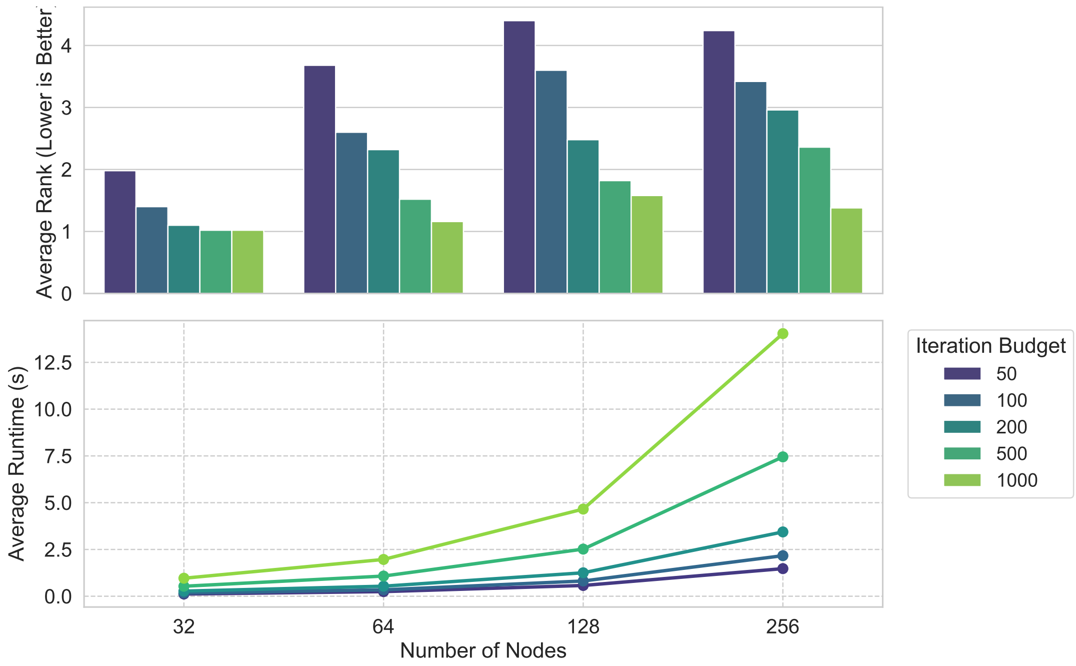

Figure S7: Analysis of optimization budget vs. performance. Top: Average rank for different iteration budgets. Bottom: Corresponding average runtime.

analysis of runtime with respect to problem size is deferred to a later section.

S1.5 Validation of Baseline Heuristics

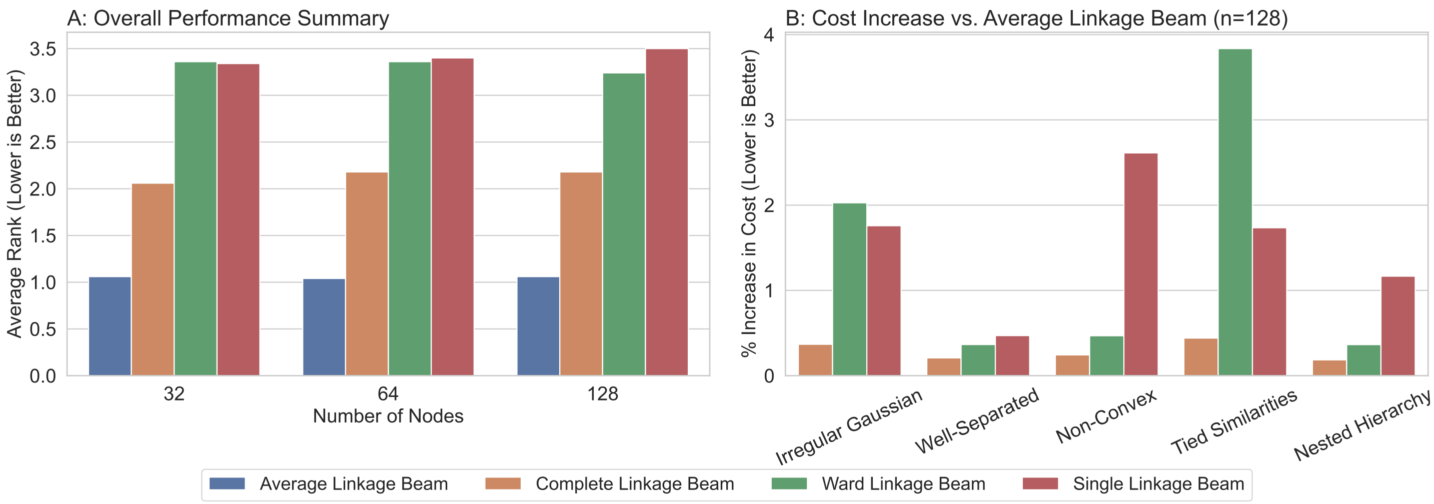

Figure S8: **Validation of the Beam Search baseline.** To ensure a rigorous comparison, we evaluated the Beam Search heuristic ($k = 100$) using four different linkage criteria: Average, Complete, Ward, and Single. **(A)** Average rank across all synthetic datasets ($n = 32, 64, 128$) shows that Average Linkage Beam (blue) consistently performs best. **(B)** Percent increase in Dasgupta's cost relative to the Average Linkage Beam baseline ($n = 128$). Positive values indicate higher (worse) costs. The Average Linkage variant consistently achieves the lowest cost across diverse data topologies, justifying its selection as the primary baseline for our comparative benchmarks.

S2 Synthetic Data Generators

Our primary synthetic benchmark is a diverse set of datasets generated to assess algorithm performance across a broad range of data topologies. The total number of samples is distributed as evenly as possible among five distinct generator types, each designed to test a specific challenge commonly found in exploratory data analysis. The goal is to create a robust test suite that evaluates a method's general performance rather than its effectiveness on a single type of data structure. The following describes the goal and high-level procedure of each generator.

Generator 1: Irregular Gaussian Cluster Generator

This generator produces datasets with classic Gaussian clusters designed to test an algorithm's robustness to common real-world conditions. The process begins by defining a random number of clusters (k) and distributing the total data points (n_{items}) among them unevenly to simulate varied cluster densities. Each cluster is generated from a multivariate Gaussian distribution with a randomized variance, creating a mix of tight and diffuse groups. To challenge methods that assume spherical shapes, a random probability exists for applying a linear transformation to each cluster's points, stretching and rotating them into various elongated shapes. Finally, a small amount of global noise is added to the entire dataset.

Generator 2: Well-Separated Spherical Cluster Generator

This generator serves as a baseline to ensure algorithms can correctly identify simple, well-defined structures. It creates a specified number of spherical clusters that are guaranteed to be well-separated in the feature space. The centers of the clusters are placed sufficiently far from one another, and the points within each cluster are drawn from a Gaussian distribution with a relatively small standard deviation. This results in dense, compact, and unambiguous cluster structures.

Generator 3: Non-Convex Shape Generator

This generator is designed to challenge algorithms that rely on assumptions of cluster convexity (e.g., that clusters are blob-like). It produces datasets with complex, non-convex topologies, including interlocking moons, concentric circles, and other non-standard shapes, using functions from the `scikit-learn` library. These datasets test an algorithm's ability to identify clusters based on local connectivity and density rather than global shape properties.

Generator 4: Discrete Data Generator for Tied Similarities

This generator produces data on a discrete grid to create a high frequency of tied similarity scores—a common scenario in real-world data (e.g., from surveys or categorical features) that can challenge deterministic clustering algorithms. Points are primarily placed around a set of cluster centers on the grid, with a certain probability of being placed randomly elsewhere. This tests an algorithm's stability and its ability to make reasonable decisions when many potential cluster merges have identical costs.

Generator 5: Nested Hierarchy Generator

This generator creates datasets with a true multi-level, nested hierarchical structure. The process starts with a single root cluster and recursively splits it into a random number of sub-clusters. At each level of the hierarchy, the centers of the child clusters are placed around the parent's center, and their variance is reduced. This creates a clear parent-child relationship,

resulting in data with distinct clusters at multiple scales. This directly tests a method's ability to recover the ground-truth hierarchical relationships.

The Combined Benchmark Suite

To create the final benchmark suite for our experiments, a master generator was used. For a given number of desired samples, it distributes the generation task as evenly as possible among the five generator types described above. For each sample, key parameters such as noise levels, cluster counts, and standard deviations are randomized within reasonable bounds. This ensures the final collection of datasets is diverse not only in type but also in its specific characteristics, providing a comprehensive and challenging test for any hierarchical clustering algorithm.

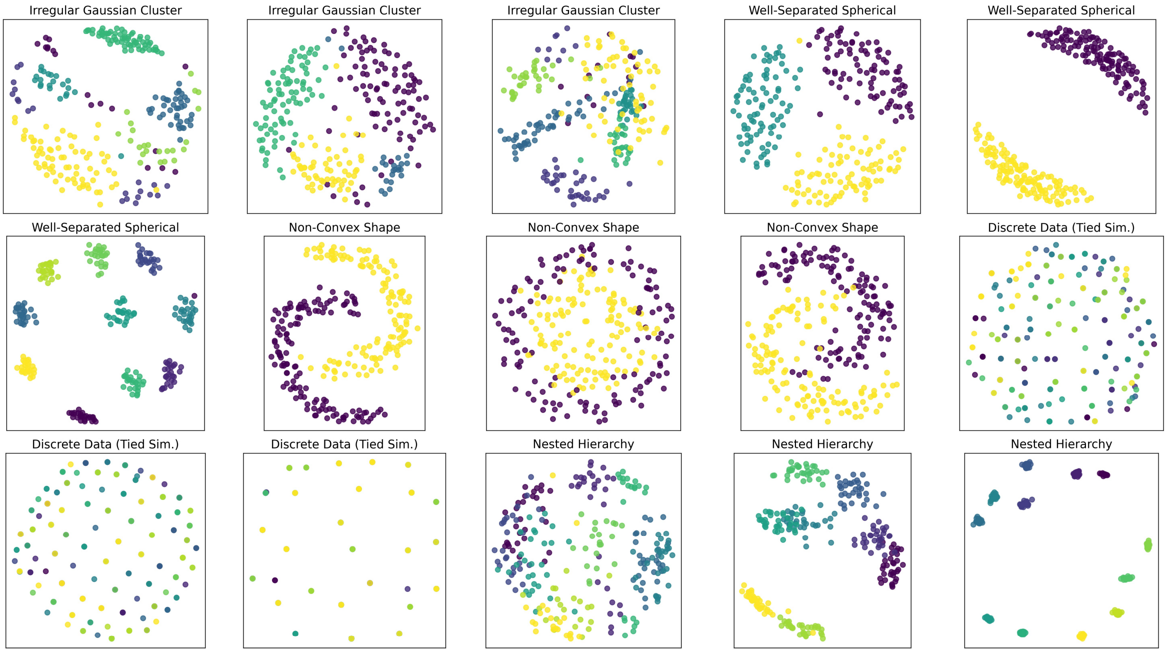

Figure S9: **Representative examples from the synthetic data benchmark.** The figure displays 15 datasets (n=256), with three samples drawn from each of the five distinct generator types. Data points are visualized in two dimensions using Multidimensional Scaling (MDS) and colored according to their ground-truth cluster assignments, illustrating the structural diversity of the benchmark suite.

S3 Runtime Analysis

To evaluate the practical scalability of our proposed method, we conducted a runtime analysis on synthetic datasets of increasing size, from n=64 to n=2048 nodes (Supplementary Figure S10). The experiments were executed on a personal computer equipped with an Intel Core i7-8565U processor, a 4-core, 8-thread CPU with a base clock of 1.80GHz. Our complete method demonstrated a practical runtime complexity of approximately $\mathcal{O}(n^2)$ within the tested range. When doubling the input size from n=1024 to n=2048, the total runtime increased by a factor of approximately 3.9x, which is consistent with quadratic scaling.

This overall quadratic behavior is primarily dictated by the cost of the surrogate-assisted optimization itself. The optimization process performs a large number of evaluations, each requiring a run of the Average Linkage algorithm. Consequently, the optimization phase has an inherent quadratic scaling that constitutes the majority of the total runtime for the tested problem sizes. The one-time cost of initial view generation — including the super-quadratic spectral method — represents a smaller, though growing, fraction of the total time, increasing from less than 1% at n=64 to 10% at n=2048. Our use of parallelization for inputs larger than n=256 effectively reduces the wall-clock time of the optimization phase but does not alter its fundamental quadratic complexity.

This analysis confirms that while DOMUS scales quadratically on the tested problem sizes due to the dominance of the $\mathcal{O}(n^2)$ optimization loop, its asymptotic complexity is bound by its most complex component. For datasets larger than those tested here, we project that the overall runtime complexity will transition from $\mathcal{O}(n^2)$ toward the super-quadratic scaling of the spectral algorithm, which will eventually become the primary performance bottleneck.

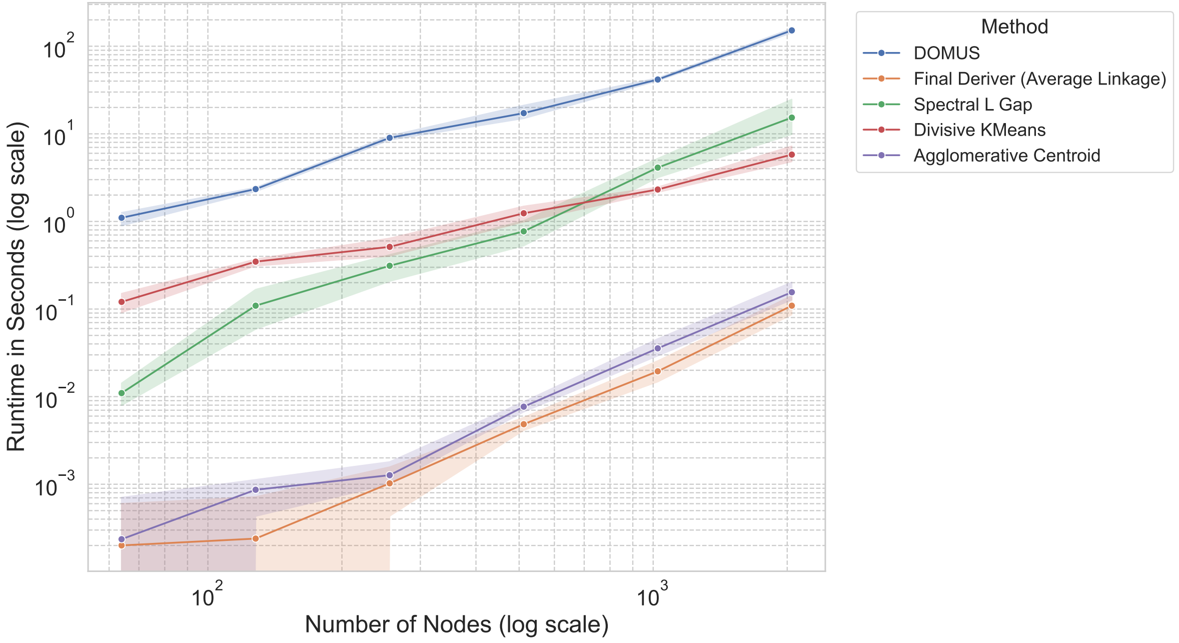

Figure S10: **Runtime analysis and scalability.** This log-log plot shows the average runtime in seconds (y-axis) as a function of problem size (x-axis, number of nodes). It compares the total runtime of the full DOMUS method against the costs of its individual components, including the three view derivers (Spectral, K-Means, Centroid) and the final Average Linkage algorithm.

S4 Additional Benchmark Results

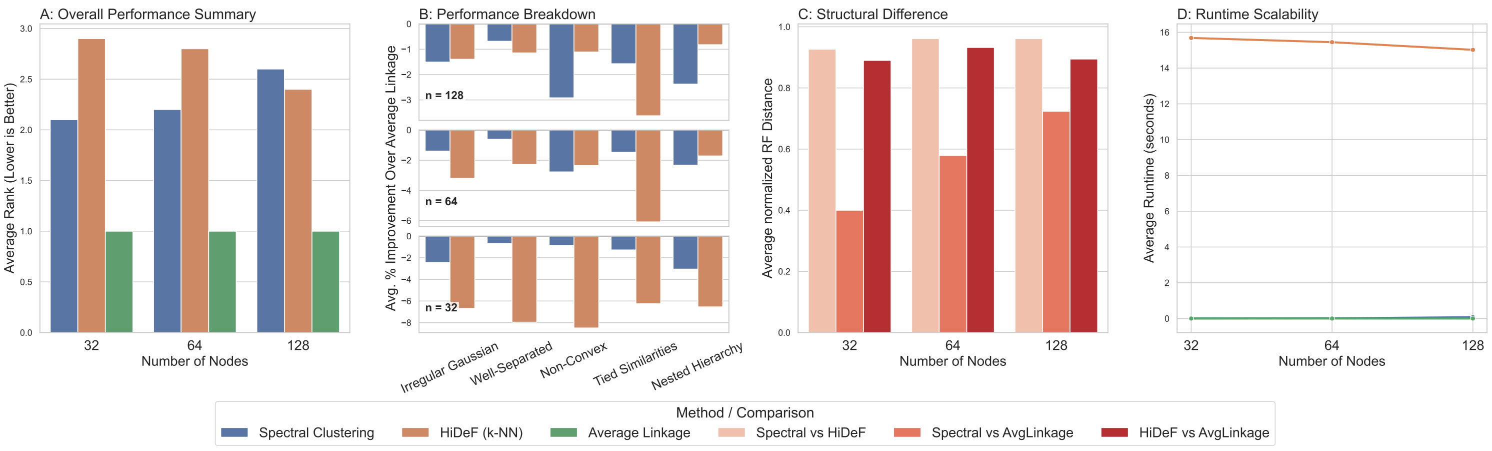

Figure S11: **Supplementary benchmark on synthetic data.** Comparison of Spectral Clustering, HiDeF, and the Average Linkage baseline, showing: **(A)** average performance rank by problem size; **(B)** percent improvement over Average Linkage by data type; **(C)** structural dissimilarity via normalized Robinson-Foulds (RF) distance; and **(D)** runtime scalability.

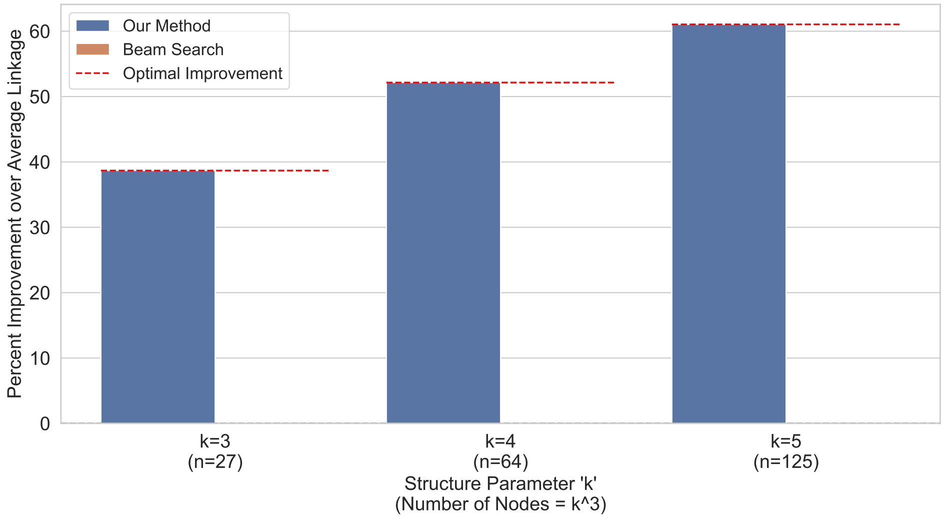

Figure S12: **Benchmark comparison on a dataset known to trap Average Linkage.** The plot shows the percent improvement over the Average Linkage baseline. DOMUS successfully avoids the local optima, matching the theoretical optimal improvement, while the Beam Search heuristic remains trapped and shows zero improvement.

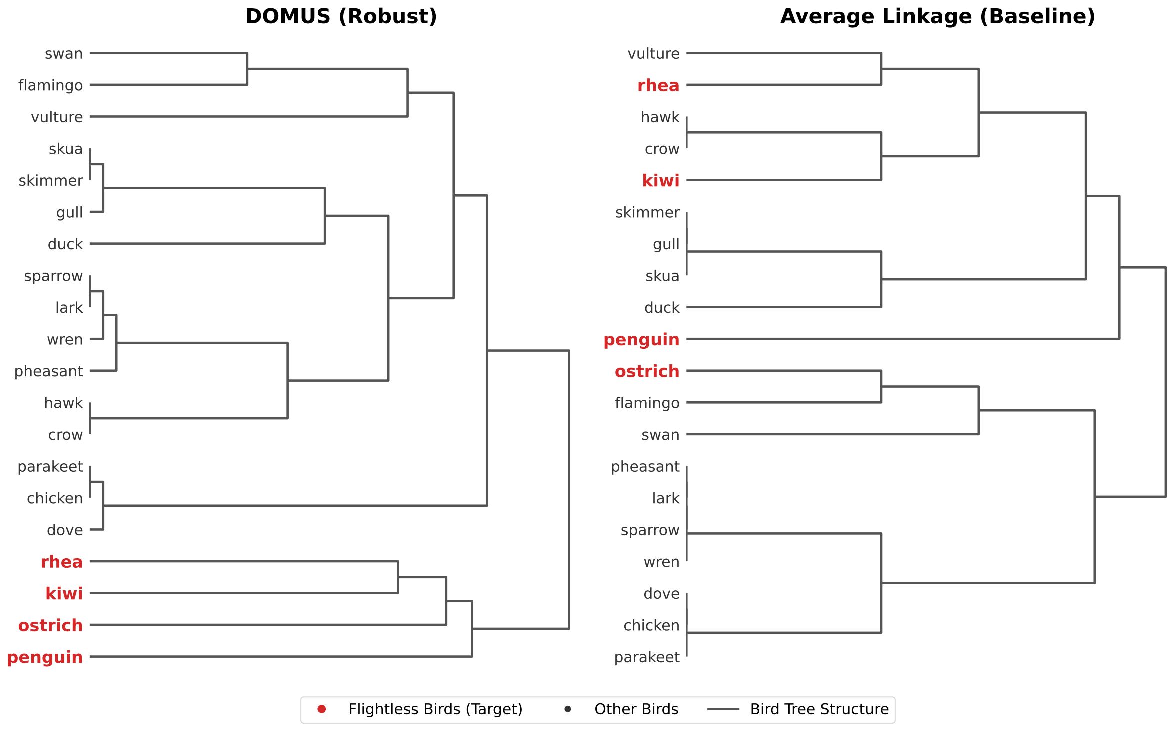

Figure S13: **Qualitative case study on the Zoo dataset.** While the global Dasgupta cost improvement on this dataset was numerically small ($\sim 0.75\%$), this figure demonstrates the significant structural impact of that optimization. **(Left)** DOMUS successfully recovers the 'flightless birds' as a single, pure clade. **(Right)** The baseline Average Linkage heuristic fails to identify this structure, scattering the species across different subtrees mixed with unrelated bird species.

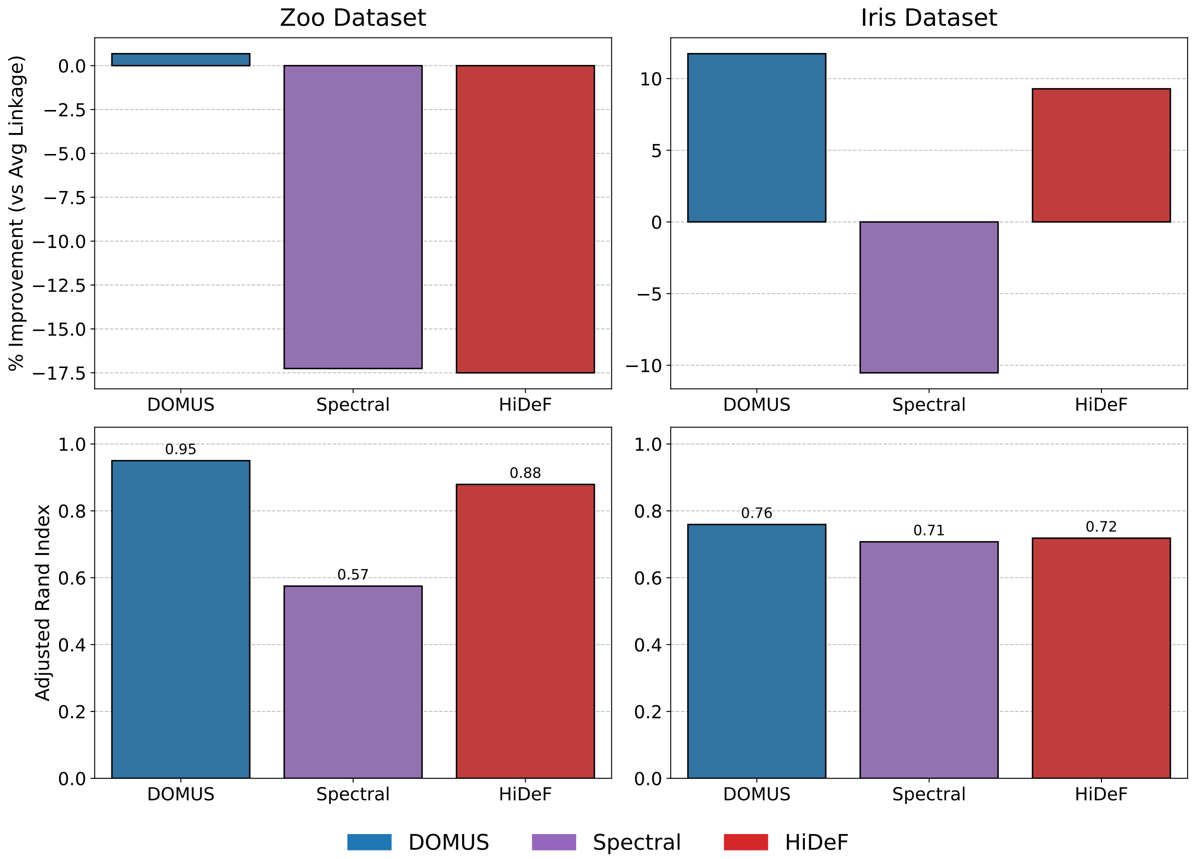

Figure S14: **Supplementary benchmark on classic datasets.** The top panels show the percent improvement in Dasgupta's cost over the Average Linkage baseline (higher is better). The bottom panels show the Adjusted Rand Index (ARI) for flat partitions obtained by cutting the dendrogram at the ground-truth number of clusters (higher is better).

S5 Cluster Stability Analysis via Jaccard Bootstrap

To assess the statistical confidence of the clusters in a final dendrogram, we employ a bootstrap validation technique. Given the computational expense of our optimization stage, we use a "fixed-parameter bootstrap" approach. This approach runs the full optimization once on the original dataset to find the optimal blending parameters, P_{orig}, under the assumption that these parameters are relatively stable to perturbations in the data. We hypothesize that these optimal parameters capture a fundamental and stable aspect of the data's structure, which should not be overly sensitive to minor perturbations from bootstrapping. For each of B bootstrap samples, the computationally inexpensive part of our pipeline is then executed by applying the fixed parameters P_{orig} to generate a new bootstrap dendrogram.

The stability of each cluster in the original dendrogram is then assessed using two metrics derived from its similarity to clusters in the bootstrap replicates:

1. **Average Jaccard Stability (AJS):** For each original cluster, we find its best-matching cluster in each bootstrap dendrogram (the one with the highest Jaccard Index). The AJS is the average of these best-match Jaccard scores over all B bootstrap samples [1]. It provides an intuitive measure of the magnitude of a cluster's stability, representing the proportion of its structure that is preserved on average.

2. **Empirical p-value:** To assess statistical significance, we generate a null distribution for each original cluster by comparing it to randomly selected clusters from the bootstrap dendrograms. For each bootstrap replicate, we sample a null cluster from the set of all $n-1$ internal nodes, specifically selecting from those with a size within 20% of the original cluster's size. The p-value is the proportion of these null Jaccard scores that are greater than or equal to the cluster's AJS, indicating the probability of observing such stability by chance.

We implemented the Jaccard-based bootstrap procedure on the Zoo and Iris datasets using 500 bootstrap iterations to assess the stability of the clusters produced by DOMUS and the Average Linkage baseline.

The analysis first served to validate the "fixed-parameter bootstrap" approach. The fact that both methods on both datasets consistently identified a large number of clusters with highly significant p-values ($p < 0.05$) provides strong evidence that the optimal blending parameters found by DOMUS are robust to data perturbations, and that the fixed-parameter shortcut is a sound approach.

The results reveal that DOMUS produces dendrograms of comparable stability to the baseline, while having achieved superior performance in recovering ground-truth structure. On both the Zoo and Iris datasets, the most stable clusters for both methods achieved Average Jaccard Stability (AJS) scores in the 0.6–0.64 range. This consistency across different data types suggests the metric is well-calibrated.

Crucially, this stability ceiling was explained by analyzing the full cluster containing all N items. Its AJS was consistently around 0.63, which corresponds to the theoretical limit for the expected Jaccard similarity of a set to its bootstrap samples, demonstrating that the most stable clusters found by DOMUS are reaching the maximum possible stability measurable by this technique.

Overall, this stability analysis provides strong evidence that our method's superior performance in recovering ground-truth structure does not come at the cost of statistical robustness. It successfully finds higher-quality hierarchies whose clusters are just as reproducible as those from the baseline method.

References

[1] Christian Hennig. “Cluster-wise assessment of cluster stability”. In: *Computational Statistics Data Analysis* 52.1 (2007), pp. 258–271. ISSN: 0167-9473. DOI: https://doi.org/10.1016/j.csda.2006.11.025. URL: https://www.sciencedirect.com/science/article/pii/S0167947306004622.
